# Iron Acquisition Mechanisms and Their Role in the Virulence of *Burkholderia* Species

**DOI:** 10.3389/fcimb.2017.00460

**Published:** 2017-11-06

**Authors:** Aaron T. Butt, Mark S. Thomas

**Affiliations:** Department of Infection, Immunity and Cardiovascular Disease, Faculty of Medicine, Dentistry and Health, University of Sheffield, Sheffield, United Kingdom

**Keywords:** *Burkholderia*, iron, siderophores, haem uptake, cystic fibrosis, melioidosis

## Abstract

*Burkholderia* is a genus within the β*-Proteobacteriaceae* that contains at least 90 validly named species which can be found in a diverse range of environments. A number of pathogenic species occur within the genus. These include *Burkholderia cenocepacia* and *Burkholderia multivorans*, opportunistic pathogens that can infect the lungs of patients with cystic fibrosis, and are members of the *Burkholderia cepacia* complex (Bcc). *Burkholderia pseudomallei* is also an opportunistic pathogen, but in contrast to Bcc species it causes the tropical human disease melioidosis, while its close relative *Burkholderia mallei* is the causative agent of glanders in horses. For these pathogens to survive within a host and cause disease they must be able to acquire iron. This chemical element is essential for nearly all living organisms due to its important role in many enzymes and metabolic processes. In the mammalian host, the amount of accessible free iron is negligible due to the low solubility of the metal ion in its higher oxidation state and the tight binding of this element by host proteins such as ferritin and lactoferrin. As with other pathogenic bacteria, *Burkholderia* species have evolved an array of iron acquisition mechanisms with which to capture iron from the host environment. These mechanisms include the production and utilization of siderophores and the possession of a haem uptake system. Here, we summarize the known mechanisms of iron acquisition in pathogenic *Burkholderia* species and discuss the evidence for their importance in the context of virulence and the establishment of infection in the host. We have also carried out an extensive bioinformatic analysis to identify which siderophores are produced by each *Burkholderia* species that is pathogenic to humans.

## The role of iron in bacteria and sources of iron within the host

Iron mainly occurs in either of two oxidation states in biological systems, Fe^2+^ and Fe^3+^ (also referred to as Fe(II) and Fe(III) or ferrous and ferric, respectively), the latter being the oxidized form that also prevails in the earth's crust, whereas the former is favored by low pH and low oxygen concentrations (Sanchez et al., 2017). It is the ability of iron to be interconverted between these two states that is the basis of many redox reactions that occur in cells (Andrews et al., 2003). For almost all species of bacteria, iron is essential as it is an important component of many proteins. It may occur as part of the haem cofactor, as in cytochromes and haem-type catalases, or as an iron-sulfur center, as in ferredoxins, rubredoxins, nitrogenase, sulfite reductase, and other iron-sulfur proteins, or as mono- or dinuclear non-haem iron that occurs in Fe-dependent superoxide dismutase and in class Ia ribonucleotide reductases, respectively (Caza and Kronstad, 2013). However, despite the relatively high iron content within humans and animals, it is not freely available due to sequestration by proteins that include hemoglobin, transferrin, lactoferrin, and ferritin, and the fact that it is largely present in the intracellular compartment (Skaar, 2010). This presents a problem to pathogenic microbes that demands the possession of high affinity iron capturing systems if they are to cope with the otherwise bacteriostatic environment. The reader is referred to the following reviews for a more comprehensive discussion of this subject (Nairz et al., 2010; Skaar, 2010; Parrow et al., 2013; Runyen-Janecky, 2013).

## The genus *Burkholderia*

**Figure 1:**
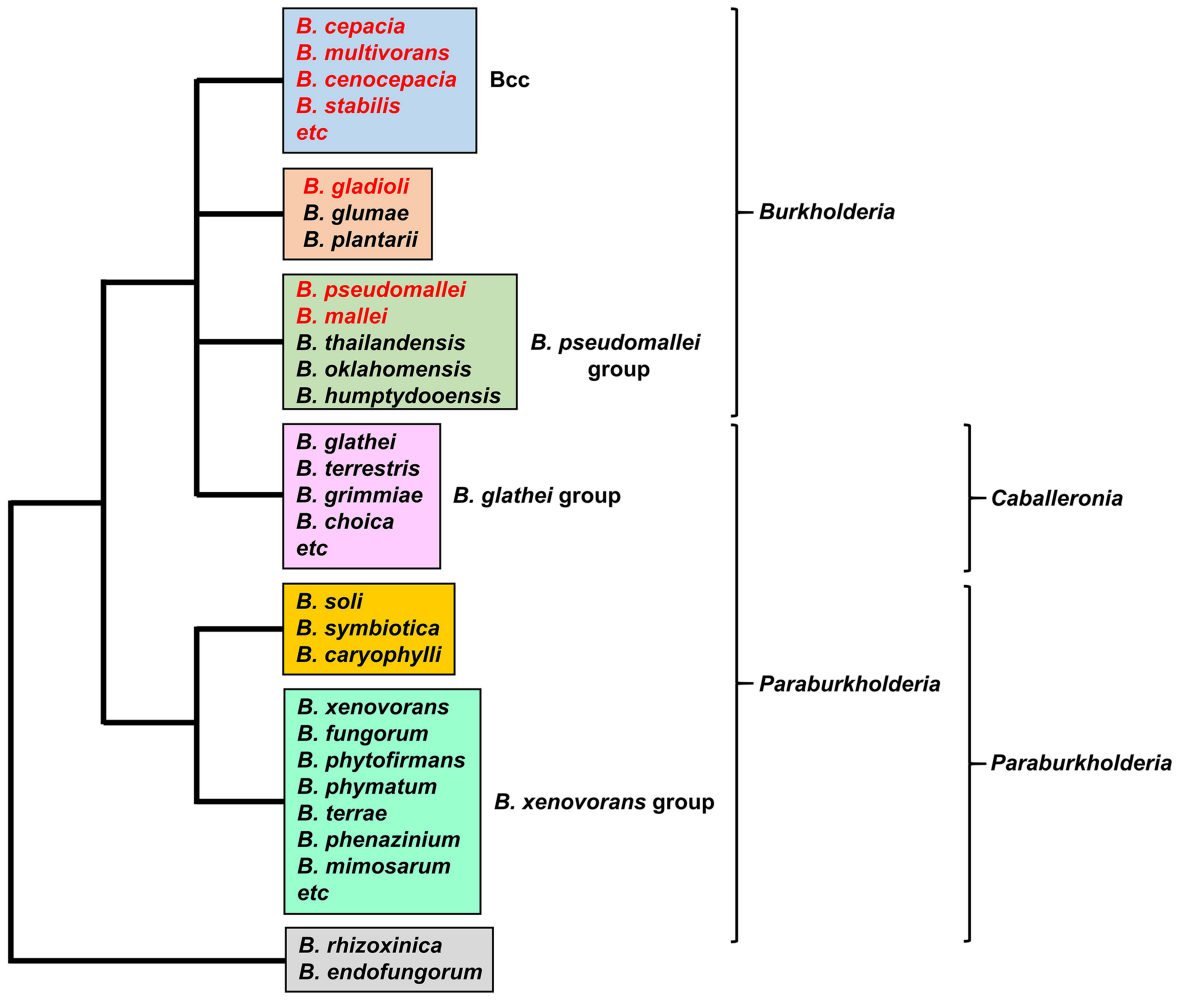
The major groups of *Burkholderia* species. Groups are based on the 16S rRNA-based phylogenetic tree (see, for example, Depoorter et al., 2016). Species most commonly associated with infections in humans are shown in red font (for a full list of the Bcc species see Table 1). Alternative classification schemes involving the proposed new genera *Paraburkholderia* and *Caballeronia* are also indicated (see text for details). Recently, it has been proposed that *B. rhizoxinica* should be transferred to a new, as yet unnamed genus (Beukes et al., 2017).

*Burkholderia* is a genus within the β*-Proteobacteriaceae* that contains at least 90 validly named species but will almost certainly include many more (Depoorter et al., 2016). Members of the genus are diverse and may be found as free-living species within soil or water, or in association with other hosts, including plants, fungi, animals and humans (Smith et al., 1995; Parke and Gurian-Sherman, 2001). They contain large genomes in the range of 7–9 Mb that are typically organized into two or three chromosomes. Based on 16S rRNA sequences, two major clades account for almost all of the currently described species (the endosymbionts *B. rhizoxinica* and *B. endofungorum* being the two exceptions; Figure 1). One clade consists of a large group of environmental and plant-associated species referred to as the *Burkholderia xenovorans* group, together with the deeper branching species *B. caryophylli, B. soli*, and *B. symbiotica*. The other clade consists of two large groups [the *B. glathei* group and the *B. cepacia* complex (or Bcc)] and two smaller groups (the *Burkholderia pseudomallei* group and a plant pathogenic group consisting of *Burkholderia gladioli, B. glumae*, and *B. plantarii*) (Depoorter et al., 2016). More recently, most of the non-pathogenic species (i.e., the *B. xenovorans* and *B. glathei* groups along with *B. caryophylli, B. soli*, and *B. symbiotica*) have been transferred to the new genus *Paraburkholderia* (Sawana et al., 2014; Oren and Garrity, 2015) and subsequently the *B. glathei* group has been transferred to the new genus *Caballeronia* (Dobritsa and Samadpour, 2016; Figure 1), although it is not clear whether the new classification schemes will be accepted by the scientific community (Depoorter et al., 2016). For the purposes of this review we will refer to all species as belonging to the genus *Burkholderia* (whichever classification scheme is adopted, the pathogenic species will remain within the genus *Burkholderia*). Of the pathogenic species, members of the Bcc and *B. pseudomallei* group can cause life-threatening infections in humans, and this feature will be discussed in this review in the context of their iron acquisition mechanisms. The phytopathogen *B. gladioli* also causes opportunistic infections in humans, but as little is known concerning its iron acquisition mechanisms it will not be discussed in detail.

## Bcc infections and cystic fibrosis

The Bcc constitute a group of at least 20 closely related species within the genus (Table 1). Members of the Bcc are well-known for causing infections in the lungs of cystic fibrosis (CF) patients, although they are also associated with infections of patients with chronic granulomatous disease and in individuals who are compromised for other reasons (Song et al., 2011). Although almost all Bcc species have been recovered from CF patient sputum, the most prevalent species are *B. cenocepacia* and *B. multivorans*, and consequently they have been the subject of most studies on potential virulence mechanisms (Reik et al., 2005; Drevinek and Mahenthiralingam, 2010; Zlosnik et al., 2015). Infections with Bcc have variable outcomes and may include transient or chronic asymptomatic infections, or they may cause a rapid decline in lung function which in some cases is accompanied by bacteraemia leading to death of the patient (“Cepacia syndrome;” Isles et al., 1984; Mahenthiralingam et al., 2001; Courtney et al., 2004; Jones et al., 2004). Infections with Bcc species are extremely difficult to eradicate due to their high level of intrinsic resistance to many antibiotics and biocides (Rose et al., 2009; Rhodes and Schweizer, 2016) [Note: prior to 2005, bacteria described as *B. cepacia* (and as *Pseudomonas cepacia* prior to the proposal of the genus *Burkholderia* in 1992) largely included members of related species within the Bcc that were not recognized as such at the time (Yabuuchi et al., 1992; Lipuma, 2005). This needs to be borne in mind when considering the results from some of the earlier investigations described below, particularly those in which a large number of “*P. cepacia*” or “*B. cepacia*” isolates were analyzed].

**Table 1:** Siderophore biosynthesis in human pathogenic members of the genus *Burkholderia*^a^.

**Species**	**Ornibactin^b^**	**Malleobactin^c^**	**Cepaciachelin^d^**	**Pyochelin^e^**	**Cepabactin^f^**
**BCC**^g^					
*B. ambifaria*	+	–	+^h^	–	?
*B. metallica*	+	–	+	–	?
*B. multivorans*	+	–	+^i^	–	?
*B. pseudomultivorans*	+	–	+^j^	–	?
*B. pyrrocinia*	+	–	+	–	?
*B. stagnalis*	+	–	+	–	?
*B. ubonensis*	+	–	+	–	?
Bcc ATCC 31433^k^	+	–	+	–	?
*B. anthina*	+	–	–	+	?
*B. cenocepacia*	+	–	–	+	–
*B. cepacia*	+	–	–^l^	+^m^	+
*B. lata*	+	–	–	+	?
*B. paludis*	–^n^	–	–	+	?
*B. seminalis*	+	–	–	+	?
*B. stabilis*^o^	+	–	–	+	?
*B. arboris*^p^	?	?	–	?	?
*B. contaminans*	+	–	–	–	?
*B. diffusa*	+	–	–	–	?
*B. dolosa*	+	–	–	–	?
*B. latens*	+	–	–	–	?
*B. territorii*	+	–	–	–^q^	?
*B. vietnamiensis*	+	–	–	–^r^	–
**PSEUDOMALLEI GROUP**					
*B. pseudomallei*	–	+	–	+	?
*B. mallei*	–	+	–	–	?
**OTHERS**					
*B. gladioli*	–	–	–	–	?

a *The potential of the listed species to produce each siderophore was deduced from a bioinformatic analysis of genome sequences using genes known to encode the biosynthesis of each siderophore as a search query (except for cepabactin where the biosynthetic genes remain to be identified). In some cases, the production (or not) of a siderophore by a specific strain has been demonstrated (see below and main text for details)*.

b *Ornibactin production has been confirmed in B. ambifaria, B. cenocepacia, B. cepacia, and B. vietnamiensis (Stephan et al., 1993; Meyer et al., 1995; Barelmann et al., 1996; Darling et al., 1998; Agnoli et al., 2006)*.

c *Presumed to be malleobactin E for B. pseudomallei and B. mallei based on the known structure of the B. thailandensis siderophore (Franke et al., 2015)*.

d *Cepaciachelin production has only been confirmed in B. ambifaria (Meyer et al., 1989)*.

e *B. cenocepacia, B. cepacia, B. paludis, and B. pseudomallei have been demonstrated to produce pyochelin whereas B. vietnamiensis isolates do not (Meyer et al., 1995; Darling et al., 1998; Alice et al., 2006; Kvitko et al., 2012; Ong et al., 2016)*.

f *Cepabactin has been identified in culture supernatants from two B. cepacia environmental strains (ATCC 25416 and ATCC17759) but not in environmental or clinical isolates of B. vietnamiensis [including the type strain TVV75 (LMG 10929)] (Meyer et al., 1989, 1995). It was not detected in culture supernatants of B. cenocepacia clinical isolates K56-2 and 715j (Darling et al., 1998)*.

g *Member species of the Bcc are as listed in Depoorter et al. (2016) with the addition of B. paludis (Ong et al., 2016) and a potential new member Bcc ATCC 31433 (Loveridge et al., 2017)*.

h *B. ambifaria PHP7 (LMG 11351) and the type strain AMMD (LMG 19182) have been shown to produce cepaciachelin, whereas strains MC40-6, MEX-5 and IOP40-10 do not possess the required genes (this study; Barelmann et al., 1996; Esmaeel et al., 2016)*.

i *Although, several B. multivorans strains encode the capacity to produce cepaciachelin, some (including ATCC 17616 and the type strain LMG 13010) do not (this study; Esmaeel et al., 2016)*.

j *Cepaciachelin gene cluster is present in B. pseudomultivorans strain MSMB368 but not in other strains currently in the database*.

k *Bcc ATCC 31433 is closely related to B. ubonensis, but possibly constitutes a separate species (Loveridge et al., 2017)*.

l *B. cepacia LK29 has the genetic capacity to produce cepaciachelin but other B. cepacia strains for which genome sequences are available do not (this study; Esmaeel et al., 2016)*.

m *B. cepacia ATCC 25416 (the type strain) and ATCC 17759 produce pyochelin whereas strain GG4 does not encode the capacity to produce this siderophore (Meyer et al., 1995; Deng et al., 2016; Esmaeel et al., 2016)*.

n *B. paludis encodes the ferric ornibactin transport system but not the biosynthetic apparatus. The fact that it retains OrbE may suggest recycling of the siderophore*.

o *At the time of writing, three B. stabilis complete genome sequences had been deposited in the database. The type strain (ATCC BAA-67) and FERMP-21014 contain the pyochelin biosynthesis and utilization genes on chromosome 2, whereas strain LA20W lacks these genes and carries the cepaciachelin gene cluster*.

p *Siderophore status is unknown due to unavailability of genome sequence information*.

q *B. territorii A63 contains the pyochelin gene cluster. Other strains, including the type strain, do not have it*.

r *Pyochelin was not detected in culture supernatants of clinical and environmental isolates of B. vietnamiensis (Meyer et al., 1995)*.

Cystic fibrosis (CF) is the most common autosomal recessive disorder among Caucasians. It is caused by mutations to the gene encoding the CF transmembrane conductor regulator (CFTR), which primarily functions as a gated chloride ion transporter but also regulates other apical membrane ion transporters (Davies et al., 2007). This defect leads to a more viscous lung mucus that impairs the action of the mucociliary escalator (Matsui et al., 1998, 2005; Boucher, 2007). Moreover, the high viscosity of the mucosal secretions may hinder the access of secreted cationic antimicrobial peptides from submucosal glands to the epithelial surface and may also restrict migration of neutrophils (see Doring et al., 2011; Tang et al., 2014 for reviews). Additional effects of a defective CFTR are also likely to be at play in facilitating pathogen survival in the airway surface liquid (ASL) of the CF lung (reviewed in Doring and Gulbins, 2009; Tang et al., 2014; Elborn, 2016), including increased abundance of amino acids (Barth and Pitt, 1996; Thomas et al., 2000), lowered pH (Song et al., 2006; Yoon et al., 2006), increased neutrophil-mediated oxidative stress (Kolpen et al., 2010), inflammation (Perez et al., 2007), hypoxic regions (Worlitzsch et al., 2002), and an altered iron status (see below). These phenomena conspire to make CF patients particularly susceptible to infection from a variety of bacterial, viral and fungal pathogens (Harrison, 2007).

Despite extensive research, the key virulence determinants of Bcc members that lead to establishment of an infection, persistence and morbidity in CF patients still remain to be established. Potential virulence determinants associated with the Bcc, including their ability to survive intracellularly (including within macrophages) (Lamothe and Valvano, 2008; Vergunst et al., 2010; Valvano, 2015; Mesureur et al., 2017), have been the subject of several comprehensive reviews and are not discussed here (Drevinek and Mahenthiralingam, 2010; Loutet and Valvano, 2010; Sousa et al., 2011). One of the problems in identifying the pathogenic mechanisms is that there are few candidate virulence determinants that are associated with all member species of the Bcc, and indeed even among different strains within the same species some these determinants may not be conserved. However, one trait that does appear to be required for virulence in Bcc species is the ability to acquire iron from iron depleted environments such as within the human host. A large number of studies have been carried out on the virulence strategies of the most commonly isolated bacterial pathogen from CF patients, *P. aeruginosa*, particularly in relation to its ability to colonize the CF lung and the role that iron acquisition mechanisms may play in this process. This knowledge may inform our understanding of the conditions prevailing within the CF lung and the iron acquisition mechanisms that are important for establishment of an infection by Bcc species. Where relevant, pertinent data obtained from studies on *P. aeruginosa* will be discussed in this review.

## The role of iron acquisition mechanisms in Bcc infections

### Iron availability in the CF lung

In considering the potential role of iron acquisition systems in the CF lung it is worth reviewing what we know considering the iron content of CF sputum and its bioavailability. Based on the known iron limiting environment of the ASL of the healthy lung, where iron is sequestered by lactoferrin, transferrin, and ferritin, it was long assumed that the CF lung also generated an iron-deficient environment (Drevinek et al., 2008). Indeed, the results of some investigations into the regulation of iron acquisition genes in the major CF pathogen, *Pseudomonas aeruginosa*, appeared to lend support to this contention (see below). However, measurements of the iron content of the CF lung have led to a reappraisal of this environment. It is now clear that the lungs of CF patients have, on average, a higher iron content than that of a healthy individual. For example, it was shown that the abundance of the iron storage protein, ferritin, was on average nearly 20-fold higher in the lungs of CF patients compared to healthy individuals, while there was ~50% less transferrin (Stites et al., 1998). Other investigators have confirmed the high ferritin concentrations in CF sputum (Reid et al., 2002, 2004). In a more recent investigation, the total iron content and the fractions that were in the Fe(II) and Fe(III) forms were measured in CF patients experiencing differing degrees of disease severity (Hunter et al., 2013). This showed a strong positive correlation between the iron content of CF sputum and the progression of the disease. Thus, patients with severe disease (as judged by their low FEV) had a high mean iron concentration in their sputum of 72 μM compared to those with a milder condition where the mean was 18 μM. Moreover, a substantial fraction of iron was present in the soluble ferrous form which increased in line with disease severity, such that in the most severe disease stage, ferrous iron constituted ~40% of the total iron load (Hunter et al., 2013). The increased abundance of Fe(II) in severe or late-stage CF disease is likely to be due to the reduction of Fe(III) by neutrophil-generated superoxides and stabilization of the resultant ferrous form by the increased prevalence of hypoxic zones in parts of the lung and acidification of the ASL.

Another source of iron that is more abundant in the CF lung is haem. This molecule becomes available through its release from hemoglobin which can occur following oxidation of the coordinated ferrous iron atom or following proteolysis of hemoglobin by host- or pathogen-derived proteases (Balla et al., 1993; Cosgrove et al., 2011). Lung tissue may release iron, including sources of haem, through injury due to the ravages of chronic inflammation (Reid et al., 2004). Moreover, CF patients experience a high frequency of micro-bleeds in their lung tissue that results in hemoglobin entering their ASL (Cosgrove et al., 2011). The frequency of airway bleeding in CF patients increases during pulmonary exacerbations where symptoms become more severe (Reid et al., 2009). The availability of ferrous iron and haem, particularly in the later stages of the disease, has potential implications for the iron acquisition systems that may be deployed by a colonizing pathogen. This change in our understanding of the iron status of the CF lung has led some workers to propose that the CF lung environment actually facilitates the growth of organisms such as *P. aeruginosa* (Reid et al., 2007).

### Iron acquisition mechanisms of the Bcc

Many bacteria synthesize and secrete low molecular weight, high affinity iron chelating compounds known as siderophores which they employ to capture iron from their local environment, particularly when this element is scarce (Chu et al., 2010). Due to its propensity to form poorly soluble hydroxides in solution, such as ${\text{Fe}(\text{OH})}_{2}^{+}$, an important role of the siderophore is to solubilize the ferric form of iron (Chipperfield and Ratledge, 2000; Ratledge and Dover, 2000). The affinities of some siderophores for iron are sufficiently high to allow them to obtain iron from host iron transport proteins such as lactoferrin and transferrin, but not from haem (Skaar, 2010). These compounds contain one, two, or three bidentate ligands that allow them to coordinate to a single Fe(III) ion, the predominant form of iron in aerobic environments at physiological pH. As iron forms hexavalent coordination complexes with its ligands, a single siderophore molecule containing three bidentate ligands (i.e., a hexadentate siderophore) will form a 1:1 complex with one ferric ion giving rise to an overall octahedral geometry (Neilands, 1995; Ratledge and Dover, 2000). Under iron replete conditions, synthesis of these molecules (and expression of other iron acquisition systems) is, in most cases, strongly downregulated in order to prevent cytoplasmic iron overload that may generate high levels of toxic reactive oxygen intermediates via the Fenton reaction (for reviews see Andrews et al., 2003; Cornelis et al., 2011).

**Figure 2:**
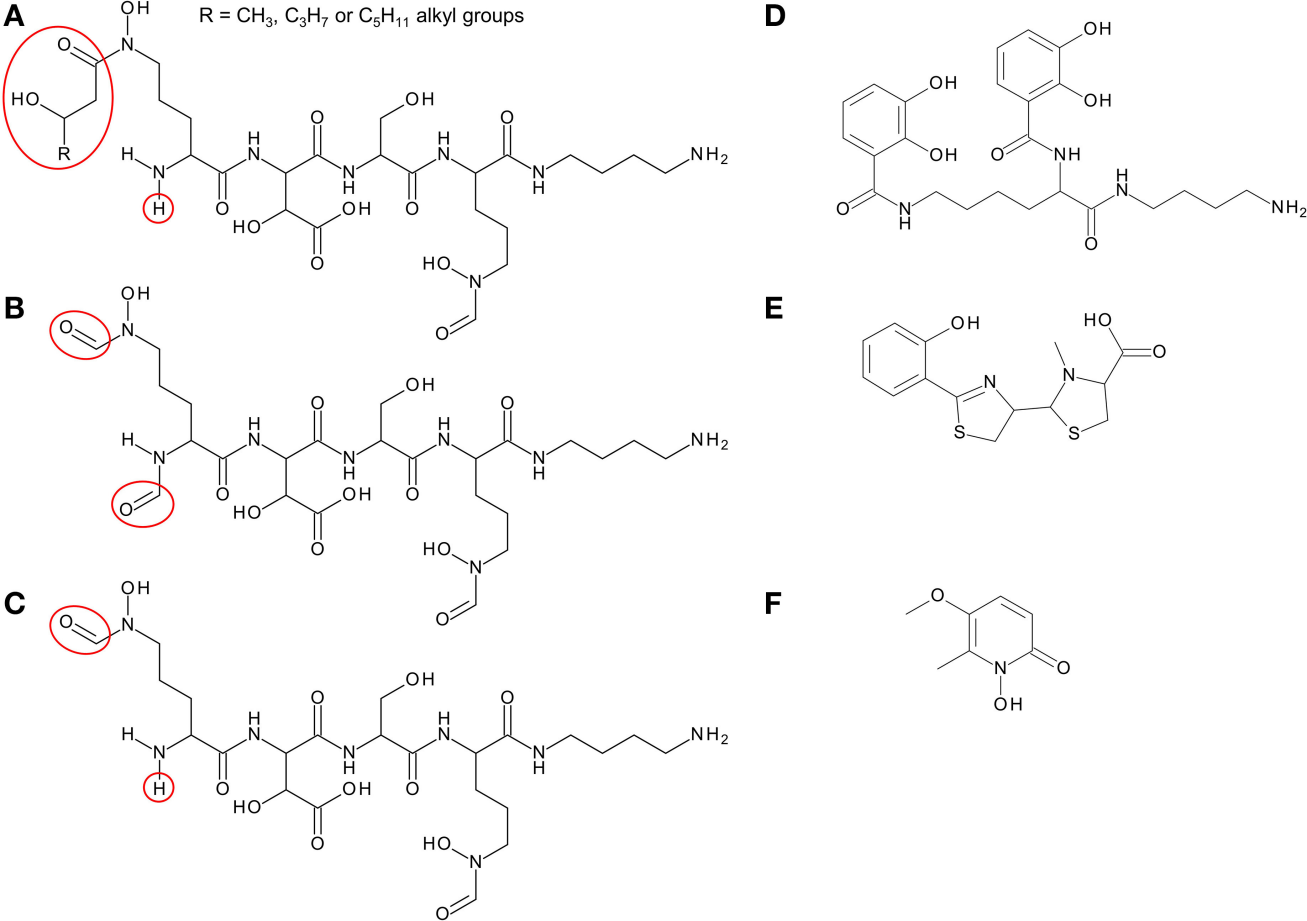
Structure of siderophores produced by *Burkholderia* species. **(A)** Ornibactins contain an N-terminal ornithine that is acylated with a C4, C6, or C8 β-hydroxycarboxylic acid on the δ-amino nitrogen atom, giving rise to ornibactin-C4, -C6, or -C8. The δ-amino nitrogen atom is also hydroxylated. The other three amino acids in the tetrapeptide are D-hydroxyaspartate, L-serine, and the C-terminal ornithine that is formylated and hydroxylated on the δ-amino nitrogen atom and the carboxyl group is conjugated to putrescine. As with the malleobactins, they contain two bidentate hydroxamate ligands and a single bidentate α-hydroxycarboxylate ligand. **(B)** Malleobactin E, the siderophore-active malleobactin congener of *B. thailandensis*. **(C)** The siderophore-active malleobactin congener of *B. xenovorans*, tentatively referred to here as “malleobactin X.” **(D)** Cepaciachelin contains two 2,3-DHBA groups that form amide linkages with the two amino groups of lysine, which in turn is conjugated to a molecule of putrescine (1,4-diaminobutane) on its α-carboxyl group. **(E)** Pyochelin contains two less commonly occurring bidentate iron-chelating groups (2-hydroxyphenyl thiazoline and N-methylthiazolidine-4-carboxylate). **(F)** Cepabactin, a cyclic hydroxamate bidentate siderophore. Chemical groups that distinguish the ornibactins and malleobactins are indicated in red circles or ellipses.

Members of the Bcc have been shown to produce one or more of four different siderophores with which they can acquire iron: ornibactin, cepaciachelin, pyochelin, and cepabactin (Meyer et al., 1989, 1995; Stephan et al., 1993; Barelmann et al., 1996; Darling et al., 1998). These siderophores include all three types (bidentate, tetradentate, and hexadentate) and all of the most common iron binding ligands are represented among them (hydroxamate, hydroxycarboxylate, catechol, and 2-hydroxyphenylthiazoline; Figure 2). The biosynthesis of pyochelin and ornibactin and the genetic regulation of their synthesis have been reviewed elsewhere, while the biosynthetic genes for cepaciachelin have been recently identified (Thomas, 2007; Esmaeel et al., 2016). For this review, we have surveyed the distribution of ornibactin, cepaciachelin, and pyochelin among the Bcc by carrying out a bioinformatic analysis of the genomes of 21 Bcc members using the corresponding biosynthetic genes as search queries (Table 1). The results accord with more limited surveys carried out previously (Deng et al., 2016; Esmaeel et al., 2016). We have also augmented the bioinformatics analysis by referencing those cases where production of a particular siderophore by specific Bcc species has actually been demonstrated. Currently, this is the only way of ascertaining which species specify cepabactin, as the biosynthetic genes remain to be identified.

**Figure 3:**
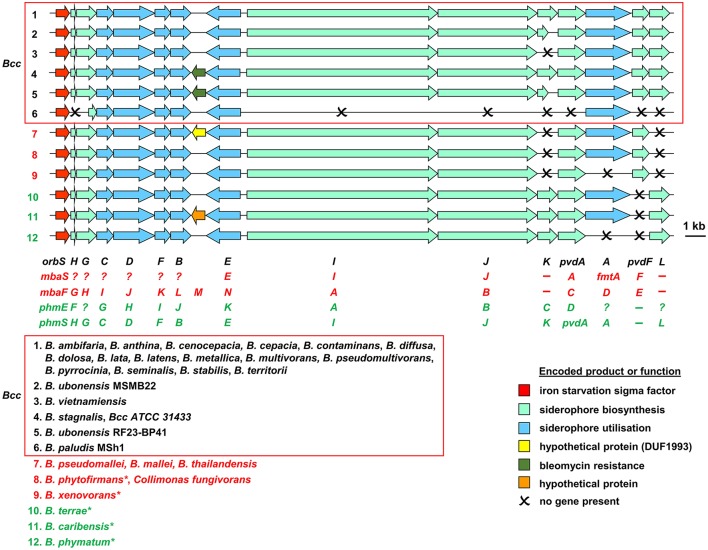
Organization of ornibactin, malleobactin, and phymabactin biosynthesis and utilization genes in pathogenic *Burkholderia* and related species. Genes are represented by block arrows and are color coded as indicated in the figure [the precise role of the MbtH-like OrbH/MbaG/PhmF proteins is unknown but they are assumed to be required for biosynthesis of the siderophore based on the requirement for other MbtH-like proteins for NRPS-mediated biosynthesis of some peptides; (Wolpert et al., 2007; Baltz, 2011)]. The numbering of each gene cluster corresponds to the numbering system for the species listed at the bottom of the figure. The font color used for each species name corresponds to the siderophore produced as follows: black, ornibactin; red, malleobactins; green, phymabactin. NRPSpredictor2 (Rottig et al., 2011) was used to predict the siderophore product based on the substrates accepted by the four adenylation domains present in OrbI/OrbJ, MbaA/MbaB, and PhmA/PhmB for each species. For systems known to specify ornibactin, the first and last (N- and C-terminal) adenylation domains of the OrbI-OrbJ NRPS pair are both predicted to accept leucine with highest probability, reflecting the presence of *N*^5^-3-hydroxyacyl-*N*^5^-hydroxyornithine and *N*^5^-formyl-*N*^5^-hydroxyornithine, respectively, at these positions in the tetrapeptide product. For malleobactin, the predicted specificity of the N-terminal adenylation domain changes to β-hydroxytyrosine although the accepted substrate is *N*^5^-formyl-*N*^5^-hydroxyornithine. In some species, such as *B. thailandensis*, the N-terminal *N*^5^-formyl-*N*^5^-hydroxyornithine is formylated on the *N*^2^-amino group upon formation of the tetrapeptide to generate malleobactin E (Franke et al., 2015), whereas in *B. xenovorans* the N-terminal *N*^5^-formyl-*N*^5^-hydroxyornithine does not appear to undergo such a tailoring reaction (Vargas-Straube et al., 2016) and so we tentatively refer to this siderophore as “malleobactin X.” The N- and C-terminal adenylation domains of PhmA-PhmB are predicted to accept aspartate and cysteine, respectively, but the structure of the product, phymabactin, is unknown, although it is predicted to have siderophore activity based on its genomic context (Esmaeel et al., 2016). In all cases, the second and third adenylation domains are predicted to accept aspartate and serine, respectively, which correspond to β-hydroxy-D-aspartate and L-serine in the final product. The nomenclature proposed for each gene is shown below the gene clusters, and the gene designations are color coded as follows: ornibactin, black (Agnoli et al., 2006); the two systems for malleobactin, red (upper, Alice et al., 2006; lower, Franke et al., 2013); the two systems for phymabactin, green (upper, Esmaeel et al., 2016; lower, this study). Genes indicated by a single letter have the same prefix as the gene name at the extreme left. Dashes indicate the absence of a gene. Question mark indicates where a gene name has not been proposed. The initial annotation of the phymabactin gene cluster (Esmaeel et al., 2016) did not include the third and last genes in the cluster or the fact that a TBDR gene occurs in other species bearing this gene cluster (upper annotation in green font). Therefore, we have proposed an alternative nomenclature based on the ornibactin gene cluster (lower annotation in green font). Scale bar refers to gene lengths and not intergenic regions, which in some cases have been exaggerated to permit alignment of each gene cluster. Species marked with an asterisk belong to a subclade within the *B. xenovorans* group and have been reassigned to the new genus *Paraburkholderia* (Sawana et al., 2014). *Collimonas* is a genus within the *Oxalobacteraceae*, a family belonging to the order *Burkholderiales*. Member species of the Bcc are enclosed in a box (*B. arboris* is not listed as its genome sequence is not currently available). Gene loci are shown in Supplementary Table 1.

Based on bioinformatic analysis of genome sequences, all Bcc species (apart from the recently described *Burkholderia paludis*) are predicted to produce the siderophore ornibactin, which is likely to act as the primary secreted iron chelator in these organisms based on its hexadenticity (Table 1 and Figure 3). Although ornibactin and malleobactin E (the siderophore produced by members of the *B. pseudomallei* group) are very similar (Figure 2), and therefore require similar biosynthetic enzymes for their assembly (Figure 3), a key feature that distinguishes the type of siderophore produced by each species is the presence of a distinct amino acid activation (adenylation) domain at the N-terminus of the larger of the two non-ribosomal peptide synthetases (NRPSs) that assemble these tetrapeptide siderophores (OrbI in the case of ornibactin). This domain activates the derivatized ornithine that will be located at the N-terminus of the tetrapeptide (for further details the reader is referred to Thomas, 2007 and the legend to Figure 3). In addition, as the δ-amino group of the N-terminal ornithine residue of ornibactin is acylated with a β-hydroxycarboxylic acid (rather than formic acid as in malleobactin E), the ornibactin gene cluster is distinguished by the presence of at least one of two genes (*orbK* and *orbL*) that are predicted to encode an acylase that catalyses this condensation reaction (Figure 3). Whereas *orbL* is always present, *orbK* may contain an internal deletion (as in *B. ubonensis*) or be absent from the cluster altogether (as in *B. vietnamiensis*). Although, the single reported *B. paludis* strain is an environmental isolate, it should be noted that in a survey of “*B. cepacia*” CF isolates carried out prior to the taxonomic reorganization of *B. cepacia* into separate Bcc species, two clinical strains were found not to produce detectable levels of ornibactin (Darling et al., 1998). It is not clear to which Bcc member species they belong or whether they are indeed members of the Bcc. One possibility is that these strains were capable of producing ornibactin prior to infection but this ability was lost through mutation during prolonged carriage as has been observed with respect to production of the major siderophore pyoverdine by some *P. aeruginosa* strains isolated from chronically infected CF patients (De Vos et al., 2001; Smith et al., 2006; Andersen et al., 2015).

**Figure 4:**
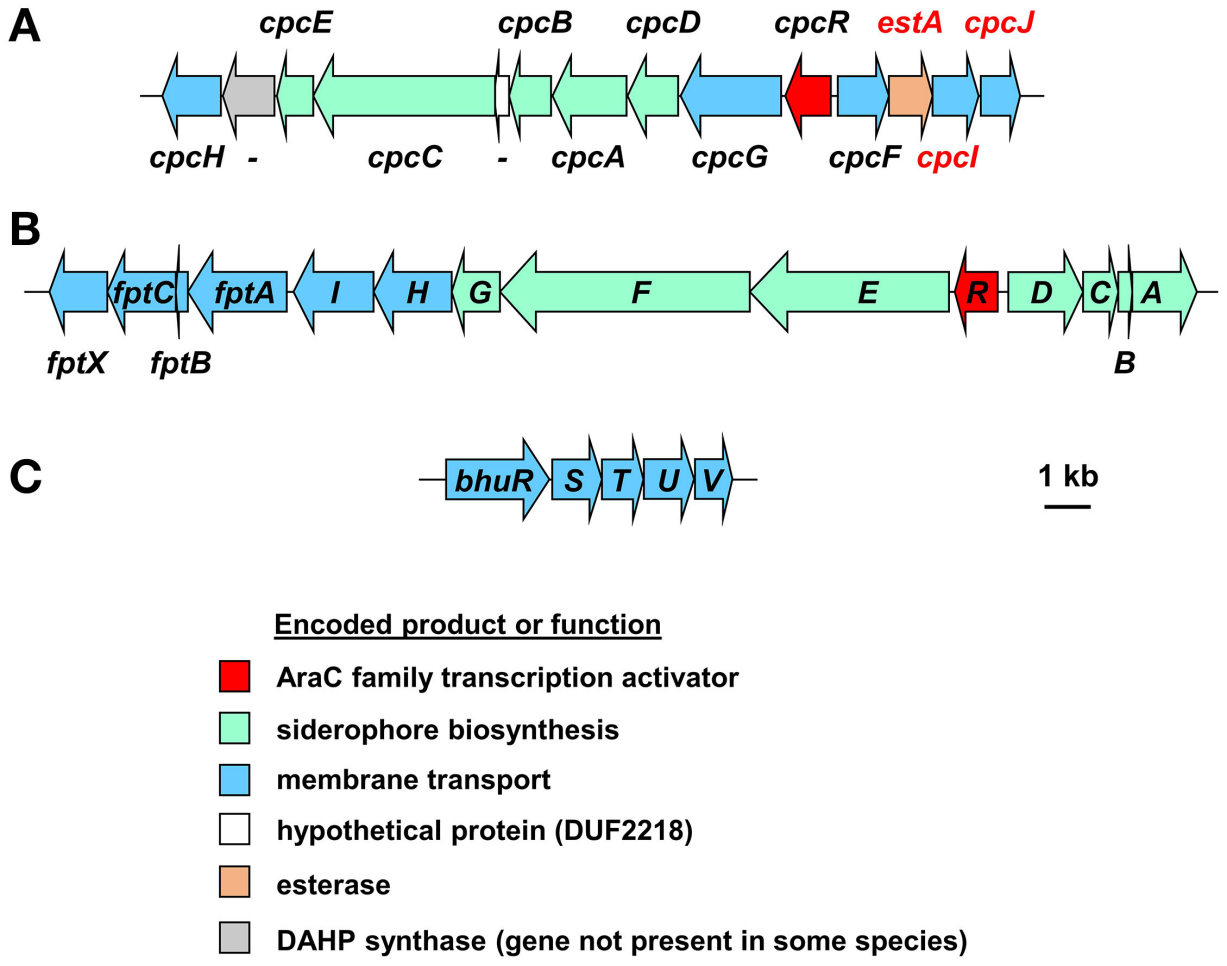
Organization of the cepaciachelin and pyochelin biosynthesis and utilization genes and the genes for the haem uptake system in *Burkholderia* species. **(A)** The cepaciachelin gene cluster in *Burkholderia* species encodes enzymes for the synthesis of the precursor 2,3-dihydroxybenzoic acid (DHBA) from chorismate and its assembly into the siderophore. Genes encoding possible cepaciachelin transport proteins are also present, including the TonB-dependent receptor, CpcG, and an MFS transporter, CpcH. Most of the genes have been previously annotated (Esmaeel et al., 2016) but the annotation has been extended here (genes labeled in red font) and includes components of a cytoplasmic membrane ABC transporter which may be involved in uptake of ferric cepaciachelin (CpcF, -I, and -J). EstA is homologous to the cytoplasmic enterobactin and salmochelin esterases Fes and IroD that are required for removal of iron from ferric-enterobactins following their uptake. However, it contains a putative Sec-dependent signal peptide and so may be periplasmically located like the *C. jejuni* enterobactin esterase, Cee (Zeng et al., 2013). This may suggest that the putative cepaciachelin receptor (CpcG) and the cytoplasmic membrane transporter also recognizes ferric-enterobactin. With the exception of *B. ambifaria* and *B. pseudomultivorans* the cepaciachelin gene cluster includes a gene encoding a DAHP synthase which catalyses the first step in the shikimate pathway that leads to the biosynthesis of chorismate from erythrose-4-phosphate and PEP. Note that in Esmaeel et al. (2016) *cphA-cphC* should be annotated as *cpcA-cpcC*, as shown here (V. Leclere, personal communication). -, gene not assigned a four letter name. Gene loci are shown in Supplementary Table 2. **(B)** The pyochelin gene cluster. Genes annotated with a single letter are designated with the prefix *pch*. Products of the biosynthetic genes generate the precursor salicylic acid from chorismate (PchAB), activate it (PchD) and assemble it into pyochelin along with two molecules of cysteine (PchCEFG). The *pchHI* and *fptABCX* genes encode membrane proteins, of which two (FptA and FptX) are involved in the transport of exogenous ferric-pyochelin across the outer and inner membranes, respectively. *fptBC* and *pchHI* appear not to be essential for export of pyochelin nor for uptake of iron-bound pyochelin (see Youard et al., 2011 for a review). Gene loci are shown in Supplementary Table 3. **(C)** Organization of the *Burkholderia* haem uptake genes, *bhuRSTUV* (Shalom et al., 2007; Thomas, 2007), also referred to as *hmuRSTUV, huvA-hmuSTUV* or *omr-hmuSTUV* (Yuhara et al., 2008; Kvitko et al., 2012; Tyrrell et al., 2015). Note that in *B. stagnalis* a VOC family protein is encoded between *bhuU* and *bhuV*, and in *B. gladioli* the *bhu* genes are organized into two operons present on separate chromosomes. Gene loci of representative species are given in Supplementary Table 4.

In addition, most Bcc species produce one or more secondary siderophores that are likely to have lower affinity for iron than ornibactin. The gene clusters specifying the biosynthesis and utilization of two of these siderophores, cepaciachelin and pyochelin, are shown in Figures 4A,B. Based on our bioinformatics survey, at least 7 species of Bcc, including *B. cenocepacia* and *B. lata*, produce pyochelin as the secondary siderophore, a feature associated with some *Pseudomonas* species (Cornelis and Matthijs, 2002), whereas in 8 other species, including some strains of *B. ambifaria* and *B. multivorans*, the secondary siderophore is cepaciachelin (Table 1). We have not identified a species possessing the genetic information required to produce both cepaciachelin and pyochelin. Both compounds are tetradentate siderophores, although the former belongs to the 2-hydroxyphenylthiazoline family whereas cepaciachelin is a bis-catecholate siderophore (Barelmann et al., 1996; Thomas, 2007; Inahashi et al., 2017). Some species do not appear to produce either of these two compounds as a secondary siderophore (Table 1). Another siderophore, the bidentate cyclic hydroxamate, cepabactin, has been detected in culture supernatants of some environmental *B. cepacia* strains in addition to ornibactin and pyochelin (see Table 1; Meyer et al., 1989, 1995). The ability of some clinical Bcc isolates of unknown taxonomic status to produce cepabactin has also been observed (Darling et al., 1998). Currently, it is not possible to infer from bioinformatics how widespread the synthesis or utilization of this siderophore is likely to be among the Bcc, although its production has not been observed in *B. cenocepacia* and *B. vietnamiensis* strains (Table 1).

**Figure 5:**
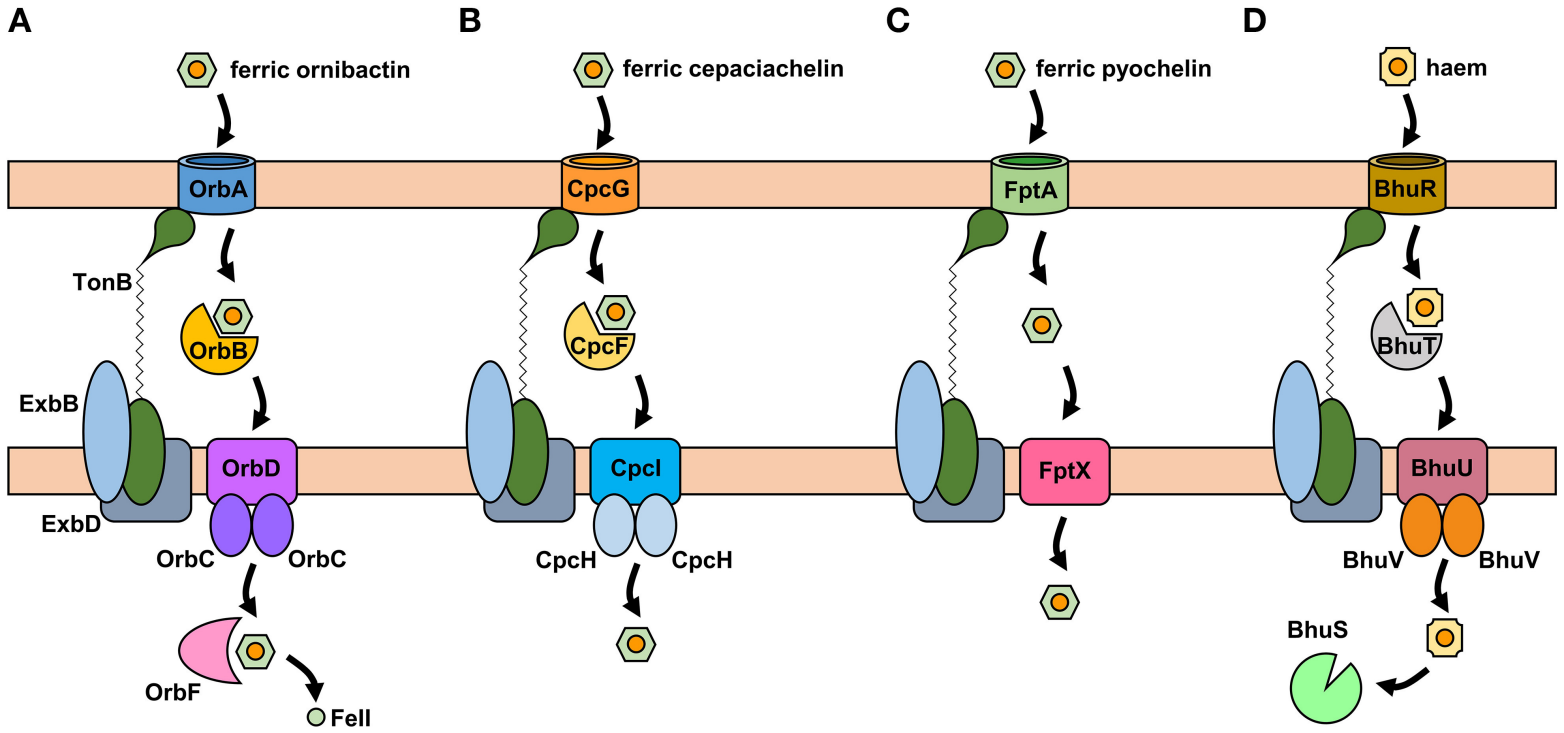
Proposed iron uptake pathways in the *Burkholderia*. **(A)** The ornibactin/malleobactin uptake system. Ferric-ornibactin is recognized by the OrbA/MbaD TBDR and is translocated into the periplasmic space through a conformational change in the plug domain of the TBDR that requires energy transduction by the TonB complex (TonB-ExbB-ExbD). The iron-siderophore complex is then transported across the cytoplasmic membrane by a periplasmic binding protein-dependent ABC transporter (OrbBCD/MbaLIJ). Oncethe ferric-siderophore complex has been internalized, iron is released from ornibactin through its reduction to the ferrous form by OrbF/MbaK. **(B)** Ferric-cepaciachelin is proposed to require the CpcG TBDR. Genes neighboring *cpcG* encode a periplasmic binding protein-dependent ABC transporter (CpcFHI) that may be involved in transport of the bis-catecholate complex across the cytoplasmic membrane. **(C)** Ferric-pyochelin uptake requires the FptA TBDR and the single subunit cytoplasmic membrane transporter, FptX. **(D)** Uptake of haem via the Bhu system. Haem uptake is proposed to follow an analogous pathway to that of ornibactin/malleobactin and cepaciachelin. Cytoplasmic haem is bound by the BhuS protein which is proposed to play a role in haem trafficking and haemostasis. If required, iron can be released form haem by haem oxygenases (not shown). The FtrABCD system is not shown. OM, outer membrane; CM, cytoplasmic membrane.

The uptake of ferric-siderophore complexes by Gram-negative bacteria such as the *Burkholderia* requires an outer membrane receptor, a 75–85 kDa polypeptide that folds into a β-barrel containing a central plug domain. Binding of a ferric-siderophore complex to the external face of the receptor triggers a conformational change in the gated receptor that allows access of the complex to the periplasmic space. The energy required for this process is derived from the proton motive force through the action of the TonB system, a complex of three different cytoplasmic membrane-anchored protein subunits: TonB, ExbB, and ExbD (Noinaj et al., 2010; Celia et al., 2016). For this reason, ferric-siderophore receptors are referred to as TonB-dependent receptors (TBDRs) or TonB-dependent transporters (TBDTs). As an example, OrbA is the TBDR for ferric-ornibactin (Figure 5A). Once the ferric-siderophore complex has entered the periplasmic space, the ferric ion is transported across the cytoplasmic membrane, either in complex with the siderophore or following release from the siderophore (depending on the system). The cytoplasmic membrane transporters are often ATP-binding cassette (ABC) transporters that consist of a periplasmic binding protein, an intrinsic membrane protein (the permease) and an ATPase located on the cytoplasmic face of the permease (Krewulak and Vogel, 2008). This type of system operates for the uptake of ferric ornibactin (Orb-B, -C, and -D) and possibly also for cepaciachelin (CpcF, -I, and -J) in the Bcc, as well as for the import of ferric malleobactin in *B. pseudomallei* and related bacteria (Figures 5A,B; Agnoli et al., 2006). In the case of ferric-pyochelin, a single subunit permease, FptX, appears to serve as the cytoplasmic membrane transporter (Figure 5C; Cuiv et al., 2004; Cunrath et al., 2015). For ferric-siderophore complexes that enter the cytoplasm, the iron is removed from the siderophore through its reduction to Fe(II), which is presumed to occur for ornibactin (Agnoli et al., 2006), or through modification or hydrolysis of the siderophore (Brickman and McIntosh, 1992; Hannauer et al., 2010).

Like many other bacterial species, *Burkholderia* spp. encode additional TBDRs that may allow them to utilize siderophores that are produced by other bacteria and fungi (“xenosiderophores”), although the potential importance of these compounds for pathogenicity is only likely to be realized in the context of a polymicrobial infection. Based on an analysis of its translated genome, *B. cenocepacia* is predicted to encode at least 20 TBDRs, many of which are likely to be involved in utilization of xenosiderophores (our unpublished results). At present, little is known concerning the nature of the xenosiderophores that can be utilized by the *Burkholderia*. However, the presence of the ornibactin transport genes in *B. paludis*, but not the biosynthetic genes, strongly suggests that ornibactin is likely to be utilized as a xenosiderophore by this species.

Members of the Bcc also specify iron acquisition systems that are not siderophore-dependent. For example, it has been shown that *B. cenocepacia* can utilize haem as an iron source (Whitby et al., 2006; Mathew et al., 2014; Tyrrell et al., 2015). *B. cenocepacia* and *B. multivorans* contain a cluster of genes (*bhuRSTUV*) that are predicted to be required for uptake of this molecule (Figure 4C; Thomas, 2007; Yuhara et al., 2008). [Note that there is currently a lack of consistency regarding the genetic nomenclature for this system in the *Burkholderia* (see below and legend to Figure 4).] Our bioinformatic survey shows that the *bhu* cluster is present on chromosome 2 in nearly all Bcc species for which whole genome sequence information is available (Supplementary Table 4). The exception is *B. vietnamiensis* which completely lacks the *bhuRSTUV* gene cluster. Although, there have not been any major studies carried out to investigate the role of this system in haem acquisition in the Bcc, this function has been established for the *bhuRSTUV* system in *B. pseudomallei* (see below), and so one can confidently infer its role in haem uptake in the Bcc. Haem appears to be an important source of iron for *P. aeruginosa* during colonization of the CF lung, and therefore one might expect members of the Bcc to take advantage of this nutrient (Konings et al., 2013). *B. cenocepacia* can also obtain iron from ferritin in a protease-dependent process (Whitby et al., 2006; Mathew et al., 2014; Tyrrell et al., 2015). Interestingly, although iron is present in the ferric form when sequestered by ferritin, siderophores are not required for uptake of ferritin-derived iron in *B. pseudomallei* (Kvitko et al., 2012).

Ferrous iron is very soluble and can passage across the outer membrane of Gram-negative bacteria through porins in an energy-independent process. However, it requires specific transporters for translocation across the cytoplasmic membrane in all bacteria. The Feo system is one such ferrous iron-specific uptake system that is present in many Gram-positive and Gram-negative bacteria (Lau et al., 2016). This system consists of a cytoplasmic membrane transport protein (FeoB) and (in most cases) a cytoplasmic component of unknown function (FeoA). In some cases, a third component (FeoC) is also present. Ferrous iron is likely to be an important source of this essential nutrient for bacteria colonizing the CF lung based on its increased abundance in the ASL of more severely afflicted CF patients. Accordingly, it has been shown that the Feo system of *P. aeruginosa* is upregulated during colonization of the lungs of every individual in a cohort of 23 infected CF patients (Konings et al., 2013). For this reason, we conducted a survey of human pathogenic *Burkholderia* species for the presence of *feoA* and *feoB*. However, only a small a minority of *B. multivorans* and *B. pseudomultivorans* strains (not the type strains), as well as the single currently identified *B. paludis* strain (MSh1), were found to encode a FeoB-like protein, although these proteins lacked a region of ~100 amino acids that is present in *P. aeruginosa* FeoB. There were no matches when FeoA was used as the query in a BLASTP search of the Bcc and *B. pseudomallei*. We conclude that the vast majority of pathogenic *Burkholderia* species lack this particular ferrous iron uptake system.

*B. cenocepacia* encodes a siderophore-independent mechanism for iron assimilation that is very similar to the FtrABCD systems reported in *Bordetella* and *Brucella* (Brickman and Armstrong, 2012; Elhassanny et al., 2013; Mathew et al., 2014). These systems share similarities with components of the *Escherichia coli* EfeUOB system that serves to import ferrous iron under aerobic conditions at low pH (Cao et al., 2007). Accordingly, in *Bordetella* spp. and *Brucella abortus*, ferrous iron is efficiently transported into these bacteria by the FtrABCD system over the pH range 6.0–7.5, in a process where the Fe(II) ion is oxidized to the ferric form using the FtrB cupredoxin component before translocation across the cytoplasmic membrane. In contrast, the *B. cenocepacia* FtrABCD system appears to utilize ferric iron as the substrate. Given the absence of a Feo system in *B. cenocepacia*, this begs the question as to the mechanism by which this species can uptake ferrous iron. *B. multivorans* also possesses an FtrABCD system, but in contrast to that of *B. cenocepacia* it does utilize ferrous iron as the substrate (S.C. Andrews, unpublished results). Therefore, it is possible that this system may play a role in ferrous iron acquisition in the CF lung for at least some Bcc species, particularly given the abundance of ferrous iron in combination with the relatively low pH of CF ASL.

Although, the substantial proportion of iron that is in the ferrous form and the increased abundance of haem in the ASL may implicate the siderophore-independent iron acquisition mechanisms of the Bcc in establishing CF lung infections, as yet, the role of these systems in the context of CF have not been investigated in any detail (see below).

### Experimental evidence for the role of iron acquisition systems in the virulence of the Bcc

#### *In vivo* studies

The earliest study on the role of iron acquisition mechanisms in the virulence of members of the Bcc was carried out prior to the discovery of the primary siderophore, ornibactin, in this group of bacteria (Sokol, 1986). Here, it was observed that from a collection of 43 “*P. cepacia”* CF isolates, ~50% produced detectable levels of pyochelin during iron limited growth *in vitro* (Pch^+^ phenotype). However, 86% of the Pch^+^ strains were associated with infections which had led to the death of the patient or were responsible for severe infections, whereas only 41% of Pch^−^ strains were associated with such outcomes. Thus, while the ability to colonize a CF patient could not be linked to the ability to produce this siderophore, there was a link between pyochelin production and the morbidity/mortality of disease in CF patients. Similarly, while addition of exogenous pyochelin to two pyochelin-negative Bcc strains did not increase bacterial numbers or bacterial persistence in infected rat lungs, it did increase the severity of infection as assessed by lung pathology (Sokol and Woods, 1988). It was proposed that the observed enhancement of lung damage brought about by exogenous addition of pyochelin to these strains was most likely due to increased dissemination of the bacteria throughout the lungs. However, it is not clear from these studies whether it was the role of pyochelin in iron acquisition that was responsible for the more severe outcome or some other effect of the siderophore. In this regard, it is known that apart from binding iron and other metals (Cuppels et al., 1987; Visca et al., 1992; Baysse et al., 2000; Braud et al., 2009) pyochelin possesses an inherent chemical reactivity that may contribute to disease severity. For example, it can promote the degradation of organotin derivatives (Sun et al., 2006), and perhaps pertinently, it can catalyse the generation of ROS such as hydroxyl radicals that result in tissue damage (Coffman et al., 1990; Britigan et al., 1994, 1997; Adler et al., 2012). The latter property is proposed to contribute to the known antibiotic activity of this siderophore (Adler et al., 2012; Ong et al., 2016). It should be noted that one important Bcc pathogen of CF patients, *B. multivorans*, does not produce pyochelin (Table 1) and the clonally related *B. cenocepacia* CF epidemic strains K56-2 and J2315 produce very little of this siderophore (see below).

A direct genotypic-phenotypic link between iron acquisition and the virulence of *B. cenocepacia* was observed during an investigation of the virulence potential of ornibactin deficient mutants in rodent models of both chronic and acute respiratory infection (Sokol et al., 1999). In this study, mutants derived from the highly transmissible epidemic *B. cenocepacia* strain, K56-2, which contained an insertionally inactivated *pvdA* gene that is required for ornibactin synthesis (Figure 3), were generated by transposon mutagenesis (*pvdA*::Tn*5*-OT182) and allelic replacement (*pvdA*::tp). Both mutants were significantly attenuated in these models. Thus, in a rat lung chronic infection model, the number of *pvdA*::Tn*5*-OT182 bacterial cells recovered from the lung was 4 logs lower than that of the wild type K56-2 strain at 28 days post infection. Furthermore, the *pvdA*::tp strain could not be recovered from the lungs after the same length of time, suggesting the infection had been cleared. The degree of pathology, as determined by the amount of inflammatory cell infiltration and exudate in the lungs, was also significantly reduced in the K56*pvdA*::tp strain compared to that of K56-2. Aerosol administration of K56-2 and the *pvdA*::Tn*5*-OT182 mutant into neutropenic mice as an acute respiratory infection model revealed that whereas the wild type strain was able to persist in the lung 7 days post infection, the *pvdA* mutant was cleared from most of the mice after 3 days. These experiments suggested the importance of ornibactin-mediated iron acquisition by the bacteria for initial colonization, persistence and resulting pathological changes within the host. However, given the difference in the iron content of the lungs of a healthy individual compared to those of a CF patient, it is not clear to what extent the conclusions from this study, which did not involve CF mice or rats, can be extrapolated to the situation in the CF lung.

While the data indicated an important role for ornibactin in lung colonization in these models, the K56-2 strain (and other members of the highly transmissible ET12 epidemic lineage) produces very low amounts of the siderophore pyochelin compared to other *B. cenocepacia* strains due to a frameshift mutation in *pchF* (Darling et al., 1998; Holden et al., 2009) (Based on perusal of the genome sequence, we presume pyochelin biosynthesis in these strains occurs through independent initiation of translation from an internal in-frame GUG codon located upstream of the frameshift site that results in production of PchF as two separate components). Thus, the role of pyochelin in the lung infection model could not be established using ET12 strains. However, given that ET12 strains cause life-threatening infections in CF patients, this would again appear to rule out an important role for pyochelin in colonization of the CF lung by *B. cenocepacia*. Later work using a *B. cenocepacia* strain (Pc715j*orbA*::tp) that produced normal amounts of pyochelin but was unable to utilize ferric-ornibactin due to disruption of the gene encoding the ferric-ornibactin TBDR, OrbA, revealed that it was cleared from rat lungs much more quickly than the WT strain (Visser et al., 2004). A ferric-pyochelin receptor mutant (Pc715j*fptA*::tp) persisted with the same efficiency as that of the WT. These data suggested that while pyochelin may have a role in the severity of infection, it is unable to compensate for the loss of a functional ornibactin utilization system. Therefore, it is ornibactin which appears to be important in order to establish an infection in this system. This may be explained by the presumed lower affinity of pyochelin than ornibactin for iron (Cox and Graham, 1979; Visca et al., 1993).

The importance of the ornibactin system for the virulence of *B. cenocepacia* has also been assessed in other infection models, including invertebrates and plants. Both a K56-2 *orbA* mutant and a K56-2 *pvdA* mutant were attenuated in the *Caenorhabditis elegans* and *Galleria mellonella* invertebrate models. The *pvdA* mutant was also slightly attenuated in the plant alfalfa model (Uehlinger et al., 2009). An *orbJ* mutant of the *B. cenocepacia* CF strain, H111, that is also deficient in the production of ornibactin, was also attenuated in the *G. mellonella* system (Mathew et al., 2014). Consistent with the virulence of the K56-2 strain in the rat lung chronic infection model, an H111 Δ*pchAB* pyochelin deficient mutant was still virulent in *G. mellonella*.

Using a modified signature-tagged mutagenesis (STM) procedure to identify genes required for survival in the rat chronic lung infection model, one of the attenuated *B. cenocepacia* K56-2 mutants which could not survive for 10 days in this model contained a transposon inserted just upstream of an ORF that encoded a haem TBDR-like protein (Hunt et al., 2004). The authors of this study indicated that the gene was the first in a cluster of genes associated with haem uptake that were located on chromosome 2, and the encoded protein was very similar to the 79 kDa RS03722 gene product of *Ralstonia solanacearum* strain GMI1000 (now reannotated as RSp0244). As RSp0244 is highly similar to BhuR we conclude that the plasposon insertion exerted polar effects on expression of the *bhuRSTUV* operon that impaired or abolished haem uptake. In contrast, genes involved in the biosynthesis and transport of ornibactin and pyochelin were not implicated in this study (Hunt et al., 2004). These observations would suggest that haem acquisition, and not siderophore-mediated iron acquisition, is an essential trait for persistence in the rat lung. Notwithstanding the different time courses of the chronic lung infection models, it is not clear why the ornibactin system should be implicated in some studies (Sokol et al., 1999; Visser et al., 2004) but not in the STM study, particularly as the ornibactin gene cluster presents a large target for plasposon-mediated disruption. One possible explanation is the selection procedure employed to construct the *B. cenocepacia* transposon mutant library. Here, mutants were selected on a mineral salts based medium in order to exclude auxotrophs. A K56-2 mutant in which the plasposon has disrupted a gene required for ornibactin synthesis or utilization would effectively be unable to obtain iron in a siderophore-dependent manner. We have observed that *B. cenocepacia* siderophore deficient mutants grow more slowly that the parental wild type strain on mineral salts medium (see for example Asghar et al., 2011), and it is possible that such mutants were omitted from the library of Hunt et al. This is not a complete explanation, however, as the haem uptake deficient mutants in the STM study can still produce ornibactin and so they might be expected to retain virulence based on other studies. This may suggest that a combination of both iron acquisition systems (haem- and ornibactin-mediated) is required for efficient colonization and persistence in the rat lung model.

Finally, the *B. cenocepacia* FtrABCD system was also investigated for a potential role in virulence in the *Galleria* wax moth model. Whereas deletion of the *ftr* system in isolation did not result in reduced virulence, when deleted in a strain that was unable to biosynthesise ornibactin and pyochelin, the mutant was more attenuated in comparison to an ornibactin-negative strain. This observation suggests that while ornibactin is the more important iron acquisition system for virulence in this model, the FtrABCD system can play a role in iron acquisition during infection in the absence of siderophores (Mathew et al., 2014).

#### *In vitro* studies

Changes in the environmental iron concentration or availability can also trigger adaptive changes in expression of virulence traits that are not directly connected to iron acquisition but rather serve other roles that contribute to survival of the bacterium under the prevailing conditions. Such examples include regulating biofilm formation in *P. aeruginosa* and capsule production in *Cryptococcus neoformans* (Singh et al., 2002; Banin et al., 2005; Jung et al., 2006). For *B. cenocepacia* it has been shown that modulating the concentration of iron causes a switch from planktonic to sessile growth. Thus, supplementing liquid cultures with ferric iron concentrations ranging from 1 to 100 μM resulted in increased levels of extracellular matrix production by strain PVI as the iron concentration was increased. Furthermore, biofilm formation induced by growth under high iron conditions resulted in more efficient invasion of A549 epithelial monolayers compared to cells grown in lower iron conditions that did not produce biofilm (Berlutti et al., 2005). In contrast, adherence of *B. cenocepacia* to A549 cells was more efficient under iron limiting conditions. The consequences of this for *B. cenocepacia* CF lung infections are not yet clear.

#### Gene expression and omics studies

Transcriptomic and proteomic analyses can be used to identify sets of genes or proteins that may be required for survival under particular conditions by virtue of their differential expression. A few such studies have been carried out with *B. cenocepacia* that have suggested an important role for iron uptake mechanisms in bacterial persistence within CF patients. However, as we discuss below, the experimental set up may not necessarily be appropriate for addressing this particular question. The first notable study looked at global gene expression during growth of *B. cenocepacia* J2315 in a basal salts medium supplemented with CF sputum in comparison to growth in unsupplemented medium (Drevinek et al., 2008). The microarray revealed upregulation of 287 genes and downregulation of 437 other genes during growth in CF sputum medium. However, only two of the upregulated genes were associated with characterized iron acquisition systems in this bacterium. These two genes (*pchR* and *pchD*) encode the transcription activator of the pyochelin gene cluster and an enzyme required for biosynthesis of the pyochelin precursor, salicylic acid, respectively, but their expression increased only 2-fold. Transcription of the ornibactin genes was not upregulated, although the gene encoding the global iron repressor, Fur, that represses the ornibactin gene cluster, was found to be downregulated 2- to 3-fold (Agnoli et al., 2006; Drevinek et al., 2008).

The authors of this work measured the iron content of their sputum medium and found it to be ~35 μM, which is more than adequate to sustain growth of *B. cenocepacia* in standard laboratory medium without upregulating siderophore biosynthesis (Drevinek et al., 2008; Madeira et al., 2013; our unpublished results). Despite the presence of CF sputum in the medium, which has been argued by some investigators to sequester iron due to the presence of various iron binding components (Wang et al., 1996; Palmer et al., 2007), their results imply that sufficient iron is available to effect repression of the ornibactin system. Accordingly, the observed induction of the *pch* genes may not be related to iron depletion but is rather a response to the presence of another component in CF sputum. It is noteworthy that genes encoding other components of the pyochelin biosynthesis machinery (particularly those enzymes required to assemble pyochelin from salicylate and cysteine) and alternative iron uptake systems (FtrABCD and BhuRSTUV) were also not upregulated. The authors contend that the CF medium they employed restricted the available iron based on the observed gross upregulation of the BCAL0270 gene, which they considered to be involved in iron acquisition (described in the study as a “ferric reductase-like transmembrane component”). However, in a basal salts medium without iron supplementation, this gene was upregulated only 2-fold compared to medium containing the standard amount of ferrous sulfate (43 μM). Moreover, this gene is currently annotated in the NCBI database as encoding a “sulfoxide reductase heme-binding subunit YedZ” and it is transcriptionally linked to BCAL0269, a gene that encodes a YedY-homologous protein. Our own bioinformatics analysis supports this annotation (results not shown). The *E. coli* YedYZ complex is a membrane anchored haem-molybdoenzyme that serves to reduce an as yet unknown S- or N-oxide (Iobbi-Nivol and Leimkuhler, 2013). Therefore, there is little evidence to support the suggestion that BCAL0270 is involved in iron acquisition. The possibility that the CF sputum medium of Drevinek et al. (2008) is iron sufficient accords with the high iron concentration included in the basal salts medium used to generate the medium. Notwithstanding the fact that the sputum is present at only 10% (w/v) in the medium, the inability of the added CF sputum to induce iron acquisition systems may suggest that its ability to sequester iron is somewhat limited and/or it is iron replete.

The results of Drevinek et al. (2008) contrast with those of Palmer et al. (2005), who monitored gene expression in the CF pathogen *P. aeruginosa* growing in a mineral salts based medium containing only CF sputum as the source of carbon and energy (“MOPS-sputum medium”), and noted that a large number of genes specifying the biosynthesis of the major siderophore, pyoverdine, as well as the entire cluster of genes specifying the biosynthesis and transport of the secondary siderophore, pyochelin, were considerably upregulated relative to their transcription in cells growing in sputum-free MOPS-glucose medium. However, although MOPS-sputum medium contained a similar amount of CF sputum to that used by Drevinek et al. (2008), the iron content (3.5 μM) was approximately one tenth of that present in the medium used in the *B. cenocepacia* experiment (in both cases iron was added as ferrous sulfate). Thus, although it should be borne in mind that two different CF pathogens are being compared, each possessing a different primary siderophore system, the amount of iron added ranged from iron replete (in the *B. cenocepacia* experiment) to a concentration that will support bacterial growth but may require a degree of upregulation of the siderophore-mediated iron acquisition system (in the *P. aeruginosa* experiment), (pyoverdine synthesis is fully repressed at ~4 μM iron and is upregulated to a progressively greater degree as concentrations of iron are decreased below 4 μM; Meyer and Abdallah, 1978). The reader may wish to consider which version of CF sputum medium more closely represents the true environment of the CF lung with respect to iron availability. The other variable at play that may influence the iron content is the source of the sputum. As discussed above, the iron content of sputum shows marked variation among CF patients, particularly in relation to the severity of the disease (Stites et al., 1998; Reid et al., 2002; Hunter et al., 2013). To illustrate the potential for a different outcome that may reflect variation in the iron content of CF sputum, in an IVET study conducted on *P. aeruginosa* growing in a mineral salts medium containing 10% CF mucus, and otherwise with no iron supplementation, only a single iron-regulated gene, *fptA* (encoding the ferric-pyochelin outer membrane receptor), was identified as being upregulated (Wang et al., 1996).

Global changes in *B. cenocepacia* gene expression have also been analyzed in a synthetic CF sputum medium (SCFM). SCFM is a defined (i.e., sputum-free) medium containing the average concentrations of ions, free amino acids, glucose and lactate as those found in the sputum of CF patients and has been shown to support similar growth rates and elicit similar changes in expression of some subsets of genes in *P. aeruginosa* to those observed during growth in MOPS-sputum medium (Palmer et al., 2007). It also contains 3.6 μM ferrous iron (i.e., similar to that of MOPS-sputum medium). Although the iron concentration of SCFM may be low enough to cause upregulation of *P. aeruginosa* siderophore gene expression relative to iron replete conditions, these genes were not (unsurprisingly) upregulated relative to cells growing in a MOPS-glucose based mineral salts medium containing an almost identical concentration of iron (Palmer et al., 2007). The contrasting high level of siderophore gene expression observed in *P. aeruginosa* growing in MOPS-sputum medium relative to cells growing in MOPS-glucose medium (Palmer et al., 2005) was rationalized on the basis that CF sputum also contains iron sequestering components which have been proposed to restrict the availability of iron (Palmer et al., 2007). Other analogous attempts to mimic CF sputum conditions using semi-synthetic media, such as ASMDM or Modified ASMDM, and comparing gene expression in *P. aeruginosa* to that in cells growing in standard laboratory media have likewise not suggested a requirement for the main siderophore-mediated iron acquisition systems in synthetic sputum-like media [apart from one case where a small (2-fold) increase in some pyochelin biosynthesis and transport genes was observed; Fung et al., 2010; Hare et al., 2012].

In apparent contrast to the observations with *P. aeruginosa*, the entire ornibactin gene cluster of *B. cenocepacia*, as well as genes encoding a number of TBDRs and a cluster of genes that encode a putative bacterioferritin-associated ferredoxin and a TonB system (BCAL2290-BCAL2293) were found to be strongly upregulated during growth of *B. cenocepacia* J2315 in SCFM in comparison to growth in soil extract medium, although the pyochelin biosynthesis genes were not upregulated in SCFM (Yoder-Himes et al., 2010). These results also contrast with those observed for *B. cenocepacia* growing in a basal salts medium supplemented with glucose, casamino acids and CF sputum (see above; Drevinek et al., 2008). The most likely reason for the latter difference is that there was a 10-fold higher concentration of iron in the CF sputum medium in comparison to SCFM. It might seem intriguing that SCFM stimulates ornibactin gene expression in *B. cenocepacia* but not expression of pyoverdine genes in *P. aeruginosa*. However, again one must proceed with caution in interpreting these data, as the fold induction of gene expression in *P. aeruginosa* cells growing in SCFM was expressed relative to medium containing the same iron concentration, whereas the comparator for the *B. cenocepacia* experiment were cells growing in soil extract medium, which has an indeterminate iron concentration. In fact, it is likely that relative to cells growing in an iron replete, nutrient rich laboratory medium, both the pyoverdine and ornibactin gene clusters may actually be upregulated in cells growing in SCFM.

To summarize the above, it is difficult to make informed judgements regarding the requirement or otherwise for various iron acquisition systems based on gene expression analysis in cells growing in defined or semi-defined media that seek to mimic CF conditions when the concentration of iron and its relative availability may not accurately reflect the situation in the patient. As an example, we note that in some more recent attempts to mimic CF conditions using synthetic media, a higher iron concentration has been used by including ferritin to better reflect the prevailing view that the iron content of the CF lung is relatively high (Hare et al., 2012). Moreover, experiments involving media which incorporate CF-derived sputa may be prone to a high degree of experimental variation according to the disease severity and consequential iron status of the sputa. Finally, obvious though this must appear, consideration of the comparator is fundamentally important in assessing whether or not iron acquisition genes are upregulated in such media.

The difficulty in reproducing CF conditions *in vitro* can be bypassed by measuring gene expression in bacterial pathogens that are present in sputum following collection of samples from CF patients. As an example, in one such study, a microarray experiment was performed using *P. aeruginosa* mRNA isolated from sputum obtained from a single patient (Son et al., 2007). In this investigation, genes specifying the biosynthesis of pyochelin were upregulated but not those encoding the biosynthesis and transport of the major siderophore pyoverdine. In a later study, an RT-qPCR analysis of gene expression in *P. aeruginosa* strains that were present in the lungs of a cohort of CF patients suggested that the siderophore pyoverdine was likely to contribute to iron acquisition in this context (Konings et al., 2013), and indeed the presence of the siderophore could be detected in CF sputa (Martin et al., 2011). In contrast, to our knowledge, studies involving direct sampling of RNA from Bcc bacteria colonizing the CF lung have not been carried out. However, sampling of *B. cenocepacia* mRNA directly from an animal model of a chronic lung infection has been carried out (O'Grady and Sokol, 2011). As discussed earlier, the ability to biosynthesise ornibactin plays an important role in chronic infections of the rat lung by *B. cenocepacia* (7 and 14 days post-infection; Visser et al., 2004). However, microarray data in which gene expression in *B. cenocepacia* K56-2 cells that were recovered from the rat lung model 3 days post-infection was compared to cells that were grown to stationary phase in a nutrient-rich broth (iron replete medium), showed no difference in ornibactin gene expression levels (O'Grady and Sokol, 2011). Therefore, the simplest interpretation of these data is that ornibactin is not required to establish an infection in this particular model system but it is required for persistence. Moreover, in contrast to the STM analysis of Hunt et al. (2004), the microarray analysis did not reveal a difference in expression of the *bhuR* and *bhuS* genes (referred to as *huvA* and *hmuS* by the authors) in the rat lung model compared to growth in iron replete medium (O'Grady and Sokol, 2011). As the STM study was conducted with animals that were infected for 10 days, one possible explanation is that haem utilization becomes important for longer term infections as has been observed in *P. aeruginosa* (see below).

Transcriptomics has been used to monitor *B. cenocepacia* adaptation to the host over time, although in this case RNA was isolated following *in vitro* culture of the bacteria. In one such study, gene expression was compared in two clonal variants that were isolated 3 years apart from a CF patient who died of cepacia syndrome. This study revealed that in the later clone, seven genes located within the ornibactin gene cluster were upregulated 1.9- to 5.9-fold compared to those in the earlier isolate when both strains were cultured on a nutrient-rich agar. Other genes potentially involved in iron uptake were also found to be more transcriptionally active in this isolate, including three genes that encode TBDRs that are not involved in ornibactin or pyochelin uptake, and two genes, *bhuR* and *bhuS* (referred to as *huvA* and *hmuS* by the authors), from the *bhuRSTUV* gene cluster that is proposed to be required for the uptake of haem (Figure 4C; Mira et al., 2011). A subsequent proteomic study employing the same pair of isolates, along with a third isolate collected just before the death of the patient, showed that the two later isolates exhibited an increased abundance of four proteins involved in siderophore-mediated iron uptake compared to the earliest clone (Madeira et al., 2013). These proteins included two TBDRs (one of which was FptA) that had increased in abundance by <2-fold, and one component of the ferric-ornibactin cytoplasmic membrane transporter (OrbC) which showed a relatively small increase in abundance (~50%) in the third isolate compared to the first. However, in contrast to the transcriptomic study, a general increase in abundance of iron acquisition proteins was not observed. Moreover, based on a CAS assay, the latter two isolates were considered to be more tolerant to low iron concentrations (siderophore production was induced at 4 or 5 μM iron, whereas in the primary isolate siderophore production was upregulated at 6 μM iron).

In a later study carried out on the same sequential clonal isolates, the upregulation of ornibactin gene expression observed in response to the iron chelating activity of exogenously added pyoverdine was significantly less pronounced in the last isolate compared to the earlier isolates (Tyrrell et al., 2015). These observations are consistent with a scenario in which selection for genetic alterations has occurred upon long term colonization of the CF lung that lead to a decreased reliance on siderophore-dependent iron acquisition by the bacterium. This may indicate a switchover to another means of iron acquisition or it may reflect a general downregulation of iron acquisition mechanisms due to increased inflammatory damage that occurs in the CF lung as the disease progresses and the consequent increased availability of iron (Cohen and Prince, 2012).

The results are analogous to those obtained from studies carried out on *P. aeruginosa*, in which it was observed that during infection of the CF lung mutations accrue in the bacterial population that result in a reduction or abolition of production of the major siderophore, pyoverdine (Marvig et al., 2014; Nguyen et al., 2014 and refs within). Evidence was also provided for increased haem usage as an iron source concomitant with a reduction in pyoverdine synthesis during the later stages of infection (Marvig et al., 2014; Nguyen et al., 2014). Consistent with this, in a separate microarray study carried out on mRNA isolated directly from a *P. aeruginosa*-infected CF patient, two genes from the pyochelin gene cluster (*pchA* and *pchC*) were observed to be upregulated 2- to 3-fold, but no pyoverdine genes were identified as being upregulated (Son et al., 2007). Although these results were obtained from work on a different CF pathogen, they are consistent with the idea that siderophore-mediated iron acquisition in the CF lung may not be of major importance to some pathogenic bacteria, particularly later on in an infection.

While these types of analyses may provide useful pointers as to the iron acquisition mechanisms that may be employed by pathogenic bacteria colonizing the CF lung, particularly if the isolate has accrued mutations that have inactivated a particular uptake system, the fact that these systems are subject to genetic regulation means that a true understanding of the iron uptake mechanisms at play during an infection will also require analysis of bacterial gene expression *in vivo*, i.e., in the CF lung.

Studies on the effects of other environmental parameters on global gene expression in *B. cenocepacia* have also been conducted that have potential implications for iron acquisition in this organism. In one investigation, the effect of oxygen depletion on gene expression was assessed, as there is evidence to suggest that within the CF lung a steep oxygen gradient is generated due to increased activity of airway epithelial Na^+^-K^+^-ATPase pumps and excessive mucin secretion. This results in the deepest layers of mucus providing a hypoxic environment that can support high densities of micro-oxic and anaerobic microbes in the CF lung (Tunney et al., 2008). In this study it was shown that *B. cenocepacia* can grow in an atmosphere with oxygen concentrations as low as 0.1% (Pessi et al., 2013). The possibility that *B. cenocepacia* may therefore occupy a low oxygen niche when colonizing the lungs of CF patients prompted an RNA-seq and shotgun proteome analysis of *B. cenocepacia* H111 growing under micro-oxic conditions (0.5% oxygen) in comparison to aerobic growth (21% oxygen). RNA-seq showed strong down regulation of the pyochelin biosynthesis genes *pchD, pchE*, and *pchF* in addition to the gene encoding the ferric-pyochelin outer membrane receptor, FptA, in micro-oxic conditions. Overall production of siderophores was also reduced in micro-oxic conditions as assessed by the CAS agar assay (Pessi et al., 2013). This kind of environment promotes the increased stabilization of Fe(II) and may favor the use of ferrous iron transport systems, such as the FtrABCD system in *B. multivorans*, over siderophore based systems.

Another environmental parameter that appears to affect the expression of iron acquisition genes that may be pertinent in the context of CF lung infections is oxidative stress. Thus, it has been observed that exposure of *B. cenocepacia* biofilms to hydrogen peroxide causes increased expression of the first few genes of the ornibactin gene cluster (*orbS, orbH*, and *orbG*; Peeters et al., 2010). While the oxygen and oxidative stress status of the niche occupied by *B. cenocepacia* in the CF lung has not yet been established, these observations bring into focus the requirement to mimic, as close as possible, the conditions of the CF lung when assessing the potential role of iron acquisition mechanisms in the virulence of respiratory pathogens.

To summarize, there appears to be a role for ornibactin-mediated iron acquisition in the virulence of *B. cenocepacia* in both vertebrate and invertebrate models of infection, including infections of the respiratory tract. However, in the context of a CF infection the role of ornibactin is less certain and there is a possibility that haem acquisition may come into play. Moreover, the severity of the disease and the particular niche that is occupied by Bcc bacteria in the lung—for example, whether it is oxygen rich or hypoxic—are also likely to dictate which iron acquisition mechanism(s) are primarily deployed. Currently, there is little evidence to support a role for the secondary siderophores in colonization and persistence by Bcc bacteria, although this is partly due to the fact that no studies on the possible roles of cepaciachelin and cepabactin have been reported. Further studies are required to establish the relative importance of the various iron acquisition mechanisms available to Bcc bacteria for colonization of the CF lung.

## *Burkholderia pseudomallei* and *Burkholderia mallei*

*B. pseudomallei* is both an environmental saprophyte and the causative agent of the tropical disease melioidosis (Wiersinga et al., 2006). This disease is endemic in South East Asia and Northern Australia with sporadic cases increasingly reported in other topical regions (Perumal Samy et al., 2017). Contraction of the disease is via cuts and abrasions, inhalation or ingestion (Wiersinga et al., 2012). Although apparently healthy individuals can become infected, conditions such as diabetes and liver disease are highly associated risk factors of melioidosis (Perumal Samy et al., 2017). The disease manifests in a range of forms from acute infections, chronic reoccurring infections, fatal sepsis or even persistent asymptomatic infections lasting for up to 60 years (White, 2003; Ngauy et al., 2005). The lung is the most commonly infected organ with the liver, spleen, skeletal muscle and prostate other sites of infection (White, 2003).

*B. mallei* is a host restricted obligate pathogen with no known environmental reservoir (Whitlock et al., 2007). This bacterium causes the zoonotic disease glanders, which is spread directly or indirectly through secretions and excretions of infected animals. This disease is chronic in horses and an acute form of the disease occurs in donkeys and mules. Infection of humans is rare and is usually the result of occupational exposure (Verma et al., 2014). *B. mallei* is considered to be a clone of *B. pseudomallei* that has undergone a process of genome reduction during host adaptation (Godoy et al., 2003; Nierman et al., 2004).

### Experimental evidence for the role of iron acquisition systems in the virulence of *B. pseudomallei*

The role of iron in *B. pseudomallei* and *B. mallei* virulence has been less well-studied than in *B. cenocepacia* mainly due to the increased hazard associated with handling these organisms which necessitates a more stringent level of containment and has also restricted the range of available selective genetic markers. Nevertheless, with the recent development of biosafety compliant tools for the genetic manipulation of these bacteria important progress has been made in our understanding of their iron acquisition systems and the role that these systems play in virulence. It is now well-established that access to iron is important for virulence by *B. pseudomallei*. For example, it has been demonstrated that the severity of *B. pseudomallei* infection of A549 macrophages and HeLa cells is increased if the cell lines are supplemented with iron. Thus, *B. pseudomallei* K96243 formed more plaques on iron-supplemented HeLa cells and invasion was significantly increased in iron-supplemented A549 cells. Furthermore, the intracellular survival of *B. pseudomallei* in A549 monolayers and the ability to induce MNGC formation, was greater when the A549 monolayers were supplemented with iron compared to non-iron supplemented controls (Amornrit et al., 2012). Interestingly, iron has also been shown to down regulate one of the type VI secretion systems (specifically T6SS-5) in *B. pseudomallei* and *B. mallei* (Burtnick and Brett, 2013). T6SS-5 is essential for virulence in hamsters and is required for multinucleated giant cell formation in infected tissue culture monolayers, a phenomenon that may facilitate cell-to-cell spread of the bacterium (Burtnick et al., 2011) [Note: T6SS-5, as designated by Shalom et al. (2007), is also referred to as T6SS-1 or the cluster 1 type VI secretion system by some authors (Schell et al., 2007; Burtnick et al., 2011)].

A limited number of studies have been carried out to determine the role of siderophores and other iron uptake systems on the virulence of *B. pseudomallei* and *B. mallei*. Both species produce the siderophore malleobactin (or more precisely, malleobactin E), that is structurally related to ornibactin (Yang et al., 1991; Alice et al., 2006; Franke et al., 2013, 2015; Figure 2). This siderophore was shown to be able to acquire iron from human transferrin and lactoferrin (Yang et al., 1993). *B. pseudomallei*, like several members of the Bcc, also produces pyochelin as a secondary siderophore (Alice et al., 2006; Kvitko et al., 2012), whereas in *B. mallei*, which has undergone extensive genome reduction, the gene cluster needed for pyochelin production is absent (Esmaeel et al., 2016). This is consistent with other evidence that pyochelin has a limited role in *Burkholderia* virulence, and loss of the ability to manufacture this siderophore in *B. mallei* may point to a more important role of pyochelin in environmental survival among other *Burkholderia* species. Moreover, *B. mallei* also produces reduced levels of malleobactin (as determined by the CAS assay) compared to *B. pseudomallei* and *B. thailandensis* which may also reflect the narrower range of niches in which it inhabits (Ong et al., 2004). *B. pseudomallei* may also secrete a third compound with iron chelating activity, although this compound has not yet been characterized (Kvitko et al., 2012).

The BPSS0240-BPSS0244 genes of *B. pseudomallei* K96243 were proposed to serve as a haem uptake system and were observed to be upregulated during growth under low iron conditions (Tuanyok et al., 2005). Based on an IVET screen, this system was shown to be induced during growth of *B. pseudomallei* NCTC 10274 within macrophages, suggesting that it may play an important role in iron acquisition during intracellular survival. In this study the authors referred to the uptake system as the Bhu (*Burkholderia* haem uptake) system based on the *Pseudomonas* Phu system (Shalom et al., 2007). By analogy with the Phu system and the related Shu system of *Shigella*, the Bhu system consists of the TBDR, BhuR (BPSS0244), a periplasmic binding protein-dependent type II ABC transporter (BhuT-BhuV) for translocation of haem across the cytoplasmic membrane, and a cytoplasmic haem binding protein (BhuS) that plays a role in haem trafficking and may also initiate haem degradation (Figure 5D; O'Neill and Wilks, 2013; Naoe et al., 2016; see Choby and Skaar, 2016) for a review. Based on a bioinformatic analysis, *B. pseudomallei* K96243 was predicted to specify two additional outer membrane receptors for haem (BPSL2724 and BPSS1742) with the former also associated with an ABC transporter system (Harland et al., 2007). However, neither of these systems shows a strong homology to characterized haem uptake systems. The BPSL2721-BPSL2724 and BPSS0240-BPSS0244 (Bhu) transport systems were later referred to as the Hem and Hmu systems, respectively, in a study conducted on strain 1710b (Kvitko et al., 2012). These authors observed that whereas deletion of the *bhu/hmu* locus compromised the ability of *B. pseudomallei* to utilize haem or hemoglobin as iron sources, deletion of the *hem* locus did not abrogate the ability to utilize haem, strongly indicating that the Bhu/Hmu system serves as the haem uptake system in this organism.

Kvitko and colleagues went on to explore the relative contributions of siderophore- and haem-mediated uptake systems to *B. pseudomallei* virulence in an acute murine melioidosis model following intranasal infection. They found that although utilization of lactoferrin-bound iron *in vitro* relied on malleobactin, inactivation of the malleobactin uptake system in strain 1710b did not cause attenuation in the murine melioidosis model (Kvitko et al., 2012). Interestingly, a strain that was defective for malleobactin, pyochelin, and haem uptake was also fully virulent in the melioidosis model, although the titres of bacteria recovered from some organs was significantly lower. As this mutant could still grow with ferritin as an iron source, it was suggested that another iron uptake system is present that could compensate for the loss of the other uptake pathways. There are at least two possibilities that could account for this. First, the CAS agar assay indicated that *B. pseudomallei* mutants lacking the ability to biosynthesise malleobactin and pyochelin specify an additional secreted iron-chelating compound of unknown identity (Kvitko et al., 2012). Secondly, *B. pseudomallei* encodes the FtrABCD system which may play a role in iron acquisition during infection (Mathew et al., 2014). Perhaps surprisingly, although *B. pseudomallei* 708a (a strain containing a >130 kb genome deletion that removes the malleobactin synthesis genes) was virulent in the mouse model used by Kvitko and colleagues, it was attenuated in *G. mellonella* (Wand et al., 2011; Kvitko et al., 2012).

The outer membrane receptors required for uptake of ferric-siderophore complexes and haem require the action of the cytoplasmic membrane-anchored TonB-ExbB-ExbD complex to energize transport of these iron sources. A *B. mallei tonB* mutant, which is unable to internalize ferric-malleobactin or haem, was shown to be completely attenuated in mice at 10^5^ CFU, in comparison to an LD_50_ of 7.4 x 10^4^ CFU for the wild type, and it also exhibited lower titres in target organs (lungs and spleen). Supplementation of the medium with ferrous iron, which is assimilated by a TonB-independent mechanism, partially restored virulence and led to higher titres of *tonB* mutant bacteria in the spleen, indicating that the inability to acquire iron is the main reason for the loss of virulence in the mutant (Mott et al., 2015). However, as TonB systems in other species have been shown to be involved in transport of other large molecules, including some enzyme cofactors (thiamine, cobalamin) and saccharides (sialic acid, sucrose, maltodextrins) the possibility exists that there are other transport requirements served by the *B. mallei* TonB system that must be met for full virulence (Schauer et al., 2008; Roy et al., 2010).

Another study in which potential novel therapeutics were screened for their ability to counteract *B. pseudomallei* killing of *C. elegans* suggested an important role for iron acquisition in virulence. Exposure of *B. pseudomallei* to the plant alkaloid curcumin (diferuloylmethane), prior to their administration to *C. elegans*, significantly enhanced the survival of the worms (Eng and Nathan, 2015). Curcumin is structurally related to bis-catecholates, which include bacterial siderophores such as azotochelin, cepaciachelin, and serratiochelin, and is itself a known iron chelator that may serve to cause a decrease in iron availability (Jiao et al., 2006). Accordingly, microarray data showed that *B. pseudomallei* treated with curcumin upregulated genes for iron transport, including those for malleobactin, pyochelin and haem uptake, as well as genes encoding the TonB system. Moreover, genes encoding the biosynthesis of malleobactin and pyochelin were upregulated and increased secretion of siderophores by treated *B. pseudomallei* was observed. The authors suggest that the anti-infective effects observed by curcumin are due to the bacteria diverting their metabolism away from virulence and toward iron uptake to ensure growth and maintenance. These observations provide suggestive evidence for the importance of iron acquisition for virulence in this organism.

## Burkholderia gladioli

*B. gladioli* was originally identified as a pathogen of *Iridaceae*, specifically irises and gladioli, and then later was recognized as the cause of infections in certain groups of immunocompromised patients, particularly among those with CF and CGD (Boyanton et al., 2005; Kennedy et al., 2007). The iron acquisition systems of this organism have yet to be determined. However, the genome sequence offers a few clues. We note that it does not contain orthologs of the genes for the biosynthesis of ornibactin, malleobactin, pyochelin, or cepaciachelin (Table 1). However, it does contain orthologs of the *bhuRSTUV* genes. Unusually, these genes are organized into two separate operons that are present on chromosome 1 (*bhuRST*) and chromosome 2 (*bhuUV*) (results not shown), but nevertheless suggest that *B. gladioli* may be able to utilize haem as an iron source during infection of human hosts. The *B. gladioli* genome also encodes a FtrABCD system. It is not known whether one or both of these systems is important for establishing an infection in humans.

## Closing remarks

To conclude, we are still far from ascertaining the relative importance of the different iron acquisition mechanisms available to the *Burkholderia* that are brought to bear during infection of a human host. A number of studies have been carried out with *B. cenocepacia* to address this question which have provided what might at first appear to be conflicting results. However, as the model systems are so varied, and the iron content and/or availability in these systems is also markedly different, we believe that most of these apparent contradictory results can be rationalized. Nonetheless, in terms of whether high affinity iron acquisition systems are essential for successful colonization of the CF lung and which ones are deployed, further studies are required. Due to the difficulty of working safely with *B. pseudomallei* and *B. mallei* fewer studies have been conducted, but it would appear that neither the known siderophores of *B. pseudomallei* nor its haem uptake system are important for systemic melioidosis. There is great scope for further work into the role of iron acquisition in the virulence of this important group of bacteria.

## Author contributions

AB and MT contributed equally to writing the manuscript. MT prepared the figures, table and Supplementary Material.

### Conflict of interest statement

The authors declare that the research was conducted in the absence of any commercial or financial relationships that could be construed as a potential conflict of interest.

## References

[B1] Adler C. Corbalan N. S. Seyedsayamdost M. R. Pomares M. F. De Cristobal R. E. Clardy J.. (2012). Catecholate siderophores protect bacteria from pyochelin toxicity. PLoS ONE 7:e46754. 10.1371/journal.pone.0046754 23071628 PMC3465284

[B2] Agnoli K. Lowe C. A. Farmer K. L. Husnain S. I. Thomas M. S. (2006). The ornibactin biosynthesis and transport genes of *Burkholderia cenocepacia* are regulated by an extracytoplasmic function sigma factor which is a part of the Fur regulon. J. Bacteriol. 188, 3631–3644. 10.1128/JB.188.10.3631-3644.2006 16672617 PMC1482860

[B3] Alice A. F. Lopez C. S. Lowe C. A. Ledesma M. A. Crosa J. H. (2006). Genetic and transcriptional analysis of the siderophore malleobactin biosynthesis and transport genes in the human pathogen *Burkholderia pseudomallei* K96243. J. Bacteriol. 188, 1551–1566. 10.1128/JB.188.4.1551-1566.2006 16452439 PMC1367220

[B4] Amornrit W. M. V. Wangteeraprasert T. Korbsrisate S. (2012). Elevated intracellular levels of iron in host cells promotes *Burkholderia pseudomallei* infection. Asian Biomed. 6, 465–471. 10.5372/1905-7415.0603.078

[B5] Andersen S. B. Marvig R. L. Molin S. Johansen H. K. Griffin A. S. (2015). Long-term social dynamics drive loss of function in pathogenic bacteria. Proc. Natl. Acad. Sci. U.S.A. 112, 10756–10761. 10.1073/pnas.1508324112 26240352 PMC4553784

[B6] Andrews S. C. Robinson A. K. Rodriguez-Quinones F. (2003). Bacterial iron homeostasis. FEMS Microbiol. Rev. 27, 215–237. 10.1016/S0168-6445(03)00055-X 12829269

[B7] Asghar A. H. Shastri S. Dave E. Wowk I. Agnoli K. Cook A. M.. (2011). The *pobA* gene of *Burkholderia cenocepacia* encodes a group I Sfp-type phosphopantetheinyltransferase required for biosynthesis of the siderophores ornibactin and pyochelin. Microbiology 157, 349–361. 10.1099/mic.0.045559-0 20966087

[B8] Balla J. Jacob H. S. Balla G. Nath K. Eaton J. W. Vercellotti G. M. (1993). Endothelial-cell heme uptake from heme proteins: induction of sensitization and desensitization to oxidant damage. Proc. Natl. Acad. Sci. U.S.A. 90, 9285–9289. 10.1073/pnas.90.20.9285 8415693 PMC47552

[B9] Baltz R. H. (2011). Function of MbtH homologs in nonribosomal peptide biosynthesis and applications in secondary metabolite discovery. J. Ind. Microbiol. Biotechnol. 38, 1747–1760. 10.1007/s10295-011-1022-8 21826462

[B10] Banin E. Vasil M. L. Greenberg E. P. (2005). Iron and *Pseudomonas aeruginosa* biofilm formation. Proc. Natl. Acad. Sci. U.S.A. 102, 11076–11081. 10.1073/pnas.0504266102 16043697 PMC1182440

[B11] Barelmann I. Meyer J. M. Taraz K. Budzikiewicz H. (1996). Cepaciachelin, a new catecholate siderophore from *Burholderia* (Pseudomonas) *cepacia*. Z. Nat. 51, 627–630.

[B12] Barth A. L. Pitt T. L. (1996). The high amino-acid content of sputum from cystic fibrosis patients promotes growth of auxotrophic *Pseudomonas aeruginosa*. J. Med. Microbiol. 45, 110–119. 10.1099/00222615-45-2-110 8683546

[B13] Baysse C. De Vos D. Naudet Y. Vandermonde A. Ochsner U. Meyer J. M.. (2000). Vanadium interferes with siderophore-mediated iron uptake in *Pseudomonas aeruginosa*. Microbiology 146 (Pt 10), 2425–2434. 10.1099/00221287-146-10-2425 11021919

[B14] Berlutti F. Morea C. Battistoni A. Sarli S. Cipriani P. Superti F.. (2005). Iron availability influences aggregation, biofilm, adhesion and invasion of Pseudomonas aeruginosa and *Burkholderia cenocepacia*. Int. J. Immunopathol. Pharmacol. 18, 661–670. 10.1177/039463200501800407 16388713

[B15] Beukes C. W. Palmer M. Manyaka P. Chan W. Y. Avontuur J. R. Van Zyl E.. (2017). Genome data provides high support for generic boundaries in *Burkholderia* sensu lato. Front Microbiol 8:1154. 10.3389/fmicb.2017.01154 28694797 PMC5483467

[B16] Boucher R. C. (2007). Airway surface dehydration in cystic fibrosis: pathogenesis and therapy. Annu. Rev. Med. 58, 157–170. 10.1146/annurev.med.58.071905.105316 17217330

[B17] Boyanton B. L. Jr. Noroski L. M. Reddy H. Dishop M. K. Hicks M. J. Versalovic J.. (2005). *Burkholderia gladioli* osteomyelitis in association with chronic granulomatous disease: case report and review. Pediatr. Infect. Dis. J. 24, 837–839. 10.1097/01.inf.0000177285.44374.dc 16148855

[B18] Braud A. Hoegy F. Jezequel K. Lebeau T. Schalk I. J. (2009). New insights into the metal specificity of the *Pseudomonas aeruginosa* pyoverdine-iron uptake pathway. Environ. Microbiol. 11, 1079–1091. 10.1111/j.1462-2920.2008.01838.x 19207567

[B19] Brickman T. J. Armstrong S. K. (2012). Iron and pH-responsive FtrABCD ferrous iron utilization system of *Bordetella* species. Mol. Microbiol. 86, 580–593. 10.1111/mmi.12003 22924881 PMC3805130

[B20] Brickman T. J. McIntosh M. A. (1992). Overexpression and purification of ferric enterobactin esterase from *Escherichia coli*. Demonstration of enzymatic hydrolysis of enterobactin and its iron complex. J. Biol. Chem. 267, 12350–12355. 1534808

[B21] Britigan B. E. Rasmussen G. T. Cox C. D. (1997). Augmentation of oxidant injury to human pulmonary epithelial cells by the *Pseudomonas aeruginosa* siderophore pyochelin. Infect. Immun. 65, 1071–1076. 9038317 10.1128/iai.65.3.1071-1076.1997 PMC175089

[B22] Britigan B. E. Serody J. S. Cohen M. S. (1994). The role of lactoferrin as an anti-inflammatory molecule. Adv. Exp. Med. Biol. 357, 143–156. 10.1007/978-1-4615-2548-6_14 7762426

[B23] Burtnick M. N. Brett P. J. (2013). *Burkholderia mallei* and *Burkholderia pseudomallei* cluster 1 type VI secretion system gene expression is negatively regulated by iron and zinc. PLoS ONE 8:e76767. 10.1371/journal.pone.0076767 24146925 PMC3795662

[B24] Burtnick M. N. Brett P. J. Harding S. V. Ngugi S. A. Ribot W. J. Chantratita N.. (2011). The cluster 1 type VI secretion system is a major virulence determinant in *Burkholderia pseudomallei*. Infect. Immun. 79, 1512–1525. 10.1128/IAI.01218-10 21300775 PMC3067527

[B25] Cao J. Woodhall M. R. Alvarez J. Cartron M. L. Andrews S. C. (2007). EfeUOB (YcdNOB) is a tripartite, acid-induced and CpxAR-regulated, low-pH Fe^2+^ transporter that is cryptic in *Escherichia coli* K-12 but functional in *E. coli* O157:H7. Mol. Microbiol. 65, 857–875. 10.1111/j.1365-2958.2007.05977.x 17627767

[B26] Caza M. Kronstad J. W. (2013). Shared and distinct mechanisms of iron acquisition by bacterial and fungal pathogens of humans. Front. Cell Infect. Microbiol. 3:80. 10.3389/fcimb.2013.00080 24312900 PMC3832793

[B27] Celia H. Noinaj N. Zakharov S. D. Bordignon E. Botos I. Santamaria M.. (2016). Structural insight into the role of the Ton complex in energy transduction. Nature 538, 60–65. 10.1038/nature19757 27654919 PMC5161667

[B28] Chipperfield J. R. Ratledge C. (2000). Salicylic acid is not a bacterial siderophore: a theoretical study. Biometals 13, 165–168. 10.1023/A:1009227206890 11016405

[B29] Choby J. E. Skaar E. P. (2016). Heme synthesis and acquisition in bacterial pathogens. J. Mol. Biol. 428, 3408–3428. 10.1016/j.jmb.2016.03.018 27019298 PMC5125930

[B30] Chu B. C. Garcia-Herrero A. Johanson T. H. Krewulak K. D. Lau C. K. Peacock R. S.. (2010). Siderophore uptake in bacteria and the battle for iron with the host; a bird's eye view. Biometals 23, 601–611. 10.1007/s10534-010-9361-x 20596754

[B31] Coffman T. J. Cox C. D. Edeker B. L. Britigan B. E. (1990). Possible role of bacterial siderophores in inflammation. Iron bound to the *Pseudomonas siderophore* pyochelin can function as a hydroxyl radical catalyst. J. Clin. Invest. 86, 1030–1037. 10.1172/JCI114805 2170442 PMC296829

[B32] Cohen T. S. Prince A. (2012). Cystic fibrosis: a mucosal immunodeficiency syndrome. Nat. Med. 18, 509–519. 10.1038/nm.2715 22481418 PMC3577071

[B33] Cornelis P. Matthijs S. (2002). Diversity of siderophore-mediated iron uptake systems in fluorescent pseudomonads: not only pyoverdines. Environ. Microbiol. 4, 787–798. 10.1046/j.1462-2920.2002.00369.x 12534462

[B34] Cornelis P. Wei Q. Andrews S. C. Vinckx T. (2011). Iron homeostasis and management of oxidative stress response in bacteria. Metallomics 3, 540–549. 10.1039/c1mt00022e 21566833

[B35] Cosgrove S. Chotirmall S. H. Greene C. M. McElvaney N. G. (2011). Pulmonary proteases in the cystic fibrosis lung induce interleukin 8 expression from bronchial epithelial cells via a heme/meprin/epidermal growth factor receptor/Toll-like receptor pathway. J. Biol. Chem. 286, 7692–7704. 10.1074/jbc.M110.183863 21193404 PMC3045023

[B36] Courtney J. M. Dunbar K. E. McDowell A. Moore J. E. Warke T. J. Stevenson M.. (2004). Clinical outcome of *Burkholderia cepacia* complex infection in cystic fibrosis adults. J. Cyst. Fibros. 3, 93–98. 10.1016/j.jcf.2004.01.005 15463892

[B37] Cox C. D. Graham R. (1979). Isolation of an iron-binding compound from *Pseudomonas aeruginosa*. J. Bacteriol. 137, 357–364. 104968 10.1128/jb.137.1.357-364.1979 PMC218458

[B38] Cuiv P. O. Clarke P. Lynch D. O'connell M. (2004). Identification of *rhtX* and *fptX*, novel genes encoding proteins that show homology and function in the utilization of the siderophores rhizobactin 1021 by *Sinorhizobium meliloti* and pyochelin by *Pseudomonas aeruginosa*, respectively. J. Bacteriol. 186, 2996–3005. 10.1128/JB.186.10.2996-3005.2004 15126460 PMC400637

[B39] Cunrath O. Gasser V. Hoegy F. Reimmann C. Guillon L. Schalk I. J. (2015). A cell biological view of the siderophore pyochelin iron uptake pathway in *Pseudomonas aeruginosa*. Environ. Microbiol. 17, 171–185. 10.1111/1462-2920.12544 24947078

[B40] Cuppels D. Stipanovic R. Stoessl A. Stothers J. (1987). The constitution and properties of a pyochelin–zinc complex. Can. J. Chem. 65, 2126–2130. 10.1139/v87-354

[B41] Darling P. Chan M. Cox A. D. Sokol P. A. (1998). Siderophore production by cystic fibrosis isolates of *Burkholderia cepacia*. Infect. Immun. 66, 874–877. 9453660 10.1128/iai.66.2.874-877.1998 PMC107988

[B42] Davies J. C. Alton E. W. F. W. Bush A. (2007). Cystic fibrosis. BMJ 335, 1255–1259. 10.1136/bmj.39391.713229.AD 18079549 PMC2137053

[B43] De Vos D. De Chial M. Cochez C. Jansen S. Tummler B. Meyer J. M.. (2001). Study of pyoverdine type and production by *Pseudomonas aeruginosa* isolated from cystic fibrosis patients: prevalence of type II pyoverdine isolates and accumulation of pyoverdine-negative mutations. Arch. Microbiol. 175, 384–388. 10.1007/s002030100278 11409549

[B44] Deng P. Wang X. Baird S. M. Showmaker K. C. Smith L. Peterson D. G. (2016). Comparative genome-wide analysis reveals that *Burkholderia contaminans* MS14 possesses multiple antimicrobial biosynthesis genes but not major genetic loci required for pathogenesis. Microbiologyopen 5, 353–369. 10.1002/mbo3.333 26769582 PMC4905989

[B45] Depoorter E. Bull M. J. Peeters C. Coenye T. Vandamme P. Mahenthiralingam E. (2016). Burkholderia: an update on taxonomy and biotechnological potential as antibiotic producers. Appl. Microbiol. Biotechnol. 100, 5215–5229. 10.1007/s00253-016-7520-x 27115756

[B46] Dobritsa A. P. Samadpour M. (2016). Transfer of eleven species of the genus *Burkholderia* to the genus *Paraburkholderia* and proposal of *Caballeronia* gen. nov. to accommodate twelve species of the genera *Burkholderia* and *Paraburkholderia*. Int. J. Syst. Evol. Microbiol. 66, 2836–2846. 10.1099/ijsem.0.001065 27054671

[B47] Doring G. Gulbins E. (2009). Cystic fibrosis and innate immunity: how chloride channel mutations provoke lung disease. Cell Microbiol. 11, 208–216. 10.1111/j.1462-5822.2008.01271.x 19068098

[B48] Doring G. Parameswaran I. G. Murphy T. F. (2011). Differential adaptation of microbial pathogens to airways of patients with cystic fibrosis and chronic obstructive pulmonary disease. FEMS Microbiol. Rev. 35, 124–146. 10.1111/j.1574-6976.2010.00237.x 20584083

[B49] Drevinek P. Mahenthiralingam E. (2010). *Burkholderia cenocepacia* in cystic fibrosis: epidemiology and molecular mechanisms of virulence. Clin. Microbiol. Infect. 16, 821–830. 10.1111/j.1469-0691.2010.03237.x 20880411

[B50] Drevinek P. Holden M. T. Ge Z. Jones A. M. Ketchell I. Gill R. T.. (2008). Gene expression changes linked to antimicrobial resistance, oxidative stress, iron depletion and retained motility are observed when *Burkholderia cenocepacia* grows in cystic fibrosis sputum. BMC Infect. Dis. 8:121. 10.1186/1471-2334-8-121 18801206 PMC2559838

[B51] Elborn J. S. (2016). Cystic fibrosis. Lancet 388, 2519–2531. 10.1016/S0140-6736(16)00576-6 27140670

[B52] Elhassanny A. E. Anderson E. S. Menscher E. A. Roop R. M. II. (2013). The ferrous iron transporter FtrABCD is required for the virulence of *Brucella abortus* 2308 in mice. Mol. Microbiol. 88, 1070–1082. 10.1111/mmi.12242 23647104

[B53] Eng S. A. Nathan S. (2015). Curcumin rescues *Caenorhabditis elegans* from a *Burkholderia pseudomallei* infection. Front. Microbiol. 6:290. 10.3389/fmicb.2015.00290 25914690 PMC4392299

[B54] Esmaeel Q. Pupin M. Kieu N. P. Chataigné G. Béchet M. Deravel J.. (2016). *Burkholderia* genome mining for nonribosomal peptide synthetases reveals a great potential for novel siderophores and lipopeptides synthesis. Microbiologyopen 5, 512–526. 10.1002/mbo3.347 27060604 PMC4906002

[B55] Franke J. Ishida K. Hertweck C. (2015). Plasticity of the malleobactin pathway and its impact on siderophore action in human pathogenic bacteria. Chemistry 21, 8010–8014. 10.1002/chem.201500757 25873483

[B56] Franke J. Ishida K. Ishida-Ito M. Hertweck C. (2013). Nitro versus hydroxamate in siderophores of pathogenic bacteria: effect of missing hydroxylamine protection in malleobactin biosynthesis. Angew. Chem. Int. Ed. Engl. 52, 8271–8275. 10.1002/anie.201303196 23821334

[B57] Fung C. Naughton S. Turnbull L. Tingpej P. Rose B. Arthur J.. (2010). Gene expression of *Pseudomonas aeruginosa* in a mucin-containing synthetic growth medium mimicking cystic fibrosis lung sputum. J. Med. Microbiol. 59, 1089–1100. 10.1099/jmm.0.019984-0 20522626

[B58] Godoy D. Randle G. Simpson A. J. Aanensen D. M. Pitt T. L. Kinoshita R.. (2003). Multilocus sequence typing and evolutionary relationships among the causative agents of melioidosis and glanders, *Burkholderia pseudomallei* and *Burkholderia mallei*. J. Clin. Microbiol. 41, 2068–2079. 10.1128/JCM.41.5.2068-2079.2003 12734250 PMC154742

[B59] Hannauer M. Barda Y. Mislin G. L. Shanzer A. Schalk I. J. (2010). The ferrichrome uptake pathway in *Pseudomonas aeruginosa* involves an iron release mechanism with acylation of the siderophore and recycling of the modified desferrichrome. J. Bacteriol. 192, 1212–1220. 10.1128/JB.01539-09 20047910 PMC2820845

[B60] Hare N. J. Solis N. Harmer C. Marzook N. B. Rose B. Harbour C.. (2012). Proteomic profiling of *Pseudomonas aeruginosa* AES-1R, PAO1 and PA14 reveals potential virulence determinants associated with a transmissible cystic fibrosis-associated strain. BMC Microbiol. 12:16. 10.1186/1471-2180-12-16 22264352 PMC3398322

[B61] Harland D. N. Dassa E. Titball R. W. Brown K. A. Atkins H. S. (2007). ATP-binding cassette systems in *Burkholderia pseudomallei* and *Burkholderia mallei*. BMC Genomics 8:83. 10.1186/1471-2164-8-83 17391530 PMC1853089

[B62] Harrison F. (2007). Microbial ecology of the cystic fibrosis lung. Microbiology 153, 917–923. 10.1099/mic.0.2006/004077-0 17379702

[B63] Holden M. T. Seth-Smith H. M. Crossman L. C. Sebaihia M. Bentley S. D. Cerdeno-Tarraga A. M.. (2009). The genome of *Burkholderia cenocepacia* J2315, an epidemic pathogen of cystic fibrosis patients. J. Bacteriol. 191, 261–277. 10.1128/JB.01230-08 18931103 PMC2612433

[B64] Hunt T. A. Kooi C. Sokol P. A. Valvano M. A. (2004). Identification of *Burkholderia cenocepacia* genes required for bacterial survival *in vivo*. Infect. Immun. 72, 4010–4022. 10.1128/IAI.72.7.4010-4022.2004 15213146 PMC427415

[B65] Hunter R. C. Asfour F. Dingemans J. Osuna B. L. Samad T. Malfroot A.. (2013). Ferrous iron is a significant component of bioavailable iron in cystic fibrosis airways. Mbio 4:e00557–13. 10.1128/mBio.00557-13 23963183 PMC3753050

[B66] Inahashi Y. Zhou S. Bibb M. J. Song L. Al-Bassam M. M. Bibb M. J.. (2017). Watasemycin biosynthesis in *Streptomyces venezuelae*: thiazoline C-methylation by a type B radical-SAM methylase homologue. Chem. Sci. 8, 2823–2831. 10.1039/C6SC03533G 28553520 PMC5427693

[B67] Iobbi-Nivol C. Leimkuhler S. (2013). Molybdenum enzymes, their maturation and molybdenum cofactor biosynthesis in *Escherichia coli*. Biochim. Biophys. Acta 1827, 1086–1101. 10.1016/j.bbabio.2012.11.007 23201473

[B68] Isles A. Maclusky I. Corey M. Gold R. Prober C. Fleming P.. (1984). *Pseudomonas cepacia* infection in cystic fibrosis: an emerging problem. J. Pediatr. 104, 206–210. 10.1016/S0022-3476(84)80993-2 6420530

[B69] Jiao Y. Wilkinson J. T. Christine Pietsch E. Buss J. L. Wang W. Planalp R.. (2006). Iron chelation in the biological activity of curcumin. Free Radic. Biol. Med. 40, 1152–1160. 10.1016/j.freeradbiomed.2005.11.003 16545682

[B70] Jones A. M. Dodd M. E. Govan J. R. Barcus V. Doherty C. J. Morris J.. (2004). *Burkholderia cenocepacia* and *Burkholderia multivorans*: influence on survival in cystic fibrosis. Thorax 59, 948–951. 10.1136/thx.2003.017210 15516469 PMC1746874

[B71] Jung W. H. Sham A. White R. Kronstad J. W. (2006). Iron regulation of the major virulence factors in the AIDS-associated pathogen *Cryptococcus neoformans*. PLoS Biol. 4:e410. 10.1371/journal.pbio.0040410 17121456 PMC1637126

[B72] Kennedy M. P. Coakley R. D. Donaldson S. H. Aris R. M. Hohneker K. Wedd J. P.. (2007). *Burkholderia gladioli:* five year experience in a cystic fibrosis and lung transplantation center. J. Cyst. Fibros. 6, 267–273. 10.1016/j.jcf.2006.10.007 17137846

[B73] Kolpen M. Hansen C. R. Bjarnsholt T. Moser C. Christensen L. D. Van Gennip M.. (2010). Polymorphonuclear leucocytes consume oxygen in sputum from chronic *Pseudomonas aeruginosa* pneumonia in cystic fibrosis. Thorax 65, 57–62. 10.1136/thx.2009.114512 19846469

[B74] Konings A. F. Martin L. W. Sharples K. J. Roddam L. F. Latham R. Reid D. W.. (2013). *Pseudomonas aeruginosa* uses multiple pathways to acquire iron during chronic infection in cystic fibrosis lungs. Infect. Immun. 81, 2697–2704. 10.1128/IAI.00418-13 23690396 PMC3719594

[B75] Krewulak K. D. Vogel H. J. (2008). Structural biology of bacterial iron uptake. Biochim. Biophys. Acta 1778, 1781–1804. 10.1016/j.bbamem.2007.07.026 17916327

[B76] Kvitko B. H. Goodyear A. Propst K. L. Dow S. W. Schweizer H. P. (2012). *Burkholderia pseudomallei* known siderophores and hemin uptake are dispensable for lethal murine melioidosis. PLoS Negl. Trop. Dis. 6:e1715. 10.1371/journal.pntd.0001715 22745846 PMC3383733

[B77] Lamothe J. Valvano M. A. (2008). *Burkholderia cenocepacia*-induced delay of acidification and phagolysosomal fusion in cystic fibrosis transmembrane conductance regulator (CFTR)-defective macrophages. Microbiology 154, 3825–3834. 10.1099/mic.0.2008/023200-0 19047750

[B78] Lau C. K. Krewulak K. D. Vogel H. J. (2016). Bacterial ferrous iron transport: the Feo system. FEMS Microbiol. Rev. 40, 273–298. 10.1093/femsre/fuv049 26684538

[B79] Lipuma J. J. (2005). Update on the *Burkholderia cepacia* complex. Curr. Opin. Pulm. Med. 11, 528–533. 10.1097/01.mcp.0000181475.85187.ed 16217180

[B80] Loutet S. A. Valvano M. A. (2010). A decade of *Burkholderia cenocepacia* virulence determinant research. Infect. Immun. 78, 4088–4100. 10.1128/IAI.00212-10 20643851 PMC2950345

[B81] Loveridge E. J. Jones C. Bull M. J. Moody S. C. Kahl M. W. Khan Z.. (2017). Reclassification of the specialized metabolite producer *Pseudomonas mesoacidophila* ATCC 31433 as a member of the *Burkholderia cepacia* complex. J. Bacteriol. 199:e00125–17. 10.1128/JB.00125-17 28439036 PMC5472815

[B82] Madeira A. Dos Santos S. C. Santos P. M. Coutinho C. P. Tyrrell J. McClean S.. (2013). Proteomic profiling of *Burkholderia cenocepacia* clonal isolates with different virulence potential retrieved from a cystic fibrosis patient during chronic lung infection. PLoS ONE 8:e83065. 10.1371/journal.pone.0083065 24349432 PMC3862766

[B83] Mahenthiralingam E. Vandamme P. Campbell M. E. Henry D. A. Gravelle A. M. Wong L. T.. (2001). Infection with *Burkholderia cepacia* complex genomovars in patients with cystic fibrosis: virulent transmissible strains of genomovar III can replace *Burkholderia multivorans*. Clin. Infect. Dis. 33, 1469–1475. 10.1086/322684 11588691

[B84] Martin L. W. Reid D. W. Sharples K. J. Lamont I. L. (2011). *Pseudomonas* siderophores in the sputum of patients with cystic fibrosis. Biometals 24, 1059–1067. 10.1007/s10534-011-9464-z 21643731

[B85] Marvig R. L. Damkiaer S. Khademi S. M. H. Markussen T. M. Molin S. Jelsbak L. (2014). Within-host evolution of *Pseudomonas aeruginosa* reveals adaptation toward iron acquisition from hemoglobin. Mbio 5:e00966-14. 10.1128/mBio.00966-14 24803516 PMC4010824

[B86] Mathew A. Eberl L. Carlier A. L. (2014). A novel siderophore-independent strategy of iron uptake in the genus *Burkholderia*. Mol. Microbiol. 91, 805–820. 10.1111/mmi.12499 24354890

[B87] Matsui H. Grubb B. R. Tarran R. Randell S. H. Gatzy J. T. Davis C. W. (1998). Evidence for periciliary liquid layer depletion, not abnormal ion composition, in the pathogenesis of cystic fibrosis airways disease. Cell 95, 1005–1015. 10.1016/S0092-8674(00)81724-9 9875854

[B88] Matsui H. Verghese M. W. Kesimer M. Schwab U. E. Randell S. H. Sheehan J. K.. (2005). Reduced three-dimensional motility in dehydrated airway mucus prevents neutrophil capture and killing bacteria on airway epithelial surfaces. J. Immunol. 175, 1090–1099. 10.4049/jimmunol.175.2.1090 16002710

[B89] Mesureur J. Feliciano J. R. Wagner N. Gomes M. C. Zhang L. Blanco-Gonzalez M. (2017). Macrophages, but not neutrophils, are critical for proliferation of *Burkholderia cenocepacia* and ensuing host-damaging inflammation. PLoS Pathog. 13:e1006437 10.1371/journal.ppat.1006437 28651010 PMC5501683

[B90] Meyer J. M. Abdallah M. A. (1978). The fluorescent pigment of pseudomonas fluorescens: biosynthesis, purification and physicochemical properties. Microbiology 107, 319–328. 10.1099/00221287-107-2-319

[B91] Meyer J. M. Hohnadel D. Halle F. (1989). Cepabactin from *Pseudomonas cepacia*, a new type of siderophore. J. Gen. Microbiol. 135, 1479–1487. 10.1099/00221287-135-6-1479 2533244

[B92] Meyer J.-M. Van Van T. Stintzi A. Berge O. Winkelmann G. (1995). Ornibactin production and transport properties in strains of *Burkholderia vietnamiensis* and *Burkholderia cepacia* (formerly *Pseudomonas cepacia*). Biometals 8, 309–317. 10.1007/BF00141604 7580051

[B93] Mira N. P. Madeira A. Moreira A. S. Coutinho C. P. Sa-Correia I. (2011). Genomic expression analysis reveals strategies of *Burkholderia cenocepacia* to adapt to cystic fibrosis patients' airways and antimicrobial therapy. PLoS ONE 6:e28831. 10.1371/journal.pone.0028831 22216120 PMC3244429

[B94] Mott T. M. Vijayakumar S. Sbrana E. Endsley J. J. Torres A. G. (2015). Characterization of the *Burkholderia mallei tonB* mutant and its potential as a backbone strain for vaccine development. PLoS Negl. Trop. Dis. 9:e0003863. 10.1371/journal.pntd.0003863 26114445 PMC4482651

[B95] Nairz M. Schroll A. Sonnweber T. Weiss G. (2010). The struggle for iron - a metal at the host-pathogen interface. Cell Microbiol. 12, 1691–1702. 10.1111/j.1462-5822.2010.01529.x 20964797

[B96] Naoe Y. Nakamura N. Doi A. Sawabe M. Nakamura H. Shiro Y.. (2016). Crystal structure of bacterial haem importer complex in the inward-facing conformation. Nat. Commun. 7:13411. 10.1038/ncomms13411 27830695 PMC5136619

[B97] Neilands J. B. (1995). Siderophores: structure and function of microbial iron transport compounds. J. Biol. Chem. 270, 26723–26726. 10.1074/jbc.270.45.26723 7592901

[B98] Ngauy V. Lemeshev Y. Sadkowski L. Crawford G. (2005). Cutaneous melioidosis in a man who was taken as a prisoner of war by the Japanese during World War II. J. Clin. Microbiol. 43, 970–972. 10.1128/JCM.43.2.970-972.2005 15695721 PMC548040

[B99] Nguyen A. T. O'Neill M. J. Watts A. M. Robson C. L. Lamont I. L. Wilks A.. (2014). Adaptation of iron homeostasis pathways by a *Pseudomonas aeruginosa* pyoverdine mutant in the cystic fibrosis lung. J. Bacteriol. 196, 2265–2276. 10.1128/JB.01491-14 24727222 PMC4054187

[B100] Nierman W. C. Deshazer D. Kim H. S. Tettelin H. Nelson K. E. Feldblyum T.. (2004). Structural flexibility in the *Burkholderia mallei* genome. Proc. Natl. Acad. Sci. U.S.A. 101, 14246–14251. 10.1073/pnas.0403306101 15377793 PMC521142

[B101] Noinaj N. Guillier M. Barnard T. J. Buchanan S. K. (2010). TonB-dependent transporters: regulation, structure, and function. Annu. Rev. Microbiol. 64, 43–60. 10.1146/annurev.micro.112408.134247 20420522 PMC3108441

[B102] O'Grady E. P. Sokol P. A. (2011). *Burkholderia cenocepacia* differential gene expression during host-pathogen interactions and adaptation to the host environment. Front. Cell. Infect. Microbiol. 1:15. 10.3389/fcimb.2011.00015 22919581 PMC3417382

[B103] O'Neill M. J. Wilks A. (2013). The *P. aeruginosa* heme binding protein PhuS is a heme oxygenase titratable regulator of heme uptake. ACS Chem. Biol. 8, 1794–1802. 10.1021/cb400165b 23947366 PMC3748626

[B104] Ong C. Ooi C. H. Wang D. Chong H. Ng K. C. Rodrigues F.. (2004). Patterns of large-scale genomic variation in virulent and avirulent *Burkholderia* species. Genome Res. 14, 2295–2307. 10.1101/gr.1608904 15520292 PMC525689

[B105] Ong K. S. Aw Y. K. Lee L. H. Yule C. M. Cheow Y. L. Lee S. M. (2016). *Burkholderia paludis* sp. nov., an antibiotic-siderophore producing novel *Burkholderia cepacia* complex species, isolated from Malaysian tropical peat swamp soil. Front. Microbiol. 7:2046. 10.3389/fmicb.2016.02046 28066367 PMC5174137

[B106] Oren A. Garrity A. G. (2015). Proposal to modify rule 27 of the international code of nomenclature of prokaryotes. Int. J. Syst. Evol. Microbiol. 65:2342. 10.1099/ijs.0.000288 25920723

[B107] Palmer K. L. Aye L. M. Whiteley M. (2007). Nutritional cues control *Pseudomonas aeruginosa* multicellular behavior in cystic fibrosis sputum. J. Bacteriol. 189, 8079–8087. 10.1128/JB.01138-07 17873029 PMC2168676

[B108] Palmer K. L. Mashburn L. M. Singh P. K. Whiteley M. (2005). Cystic fibrosis sputum supports growth and cues key aspects of *Pseudomonas aeruginosa* physiology. J. Bacteriol. 187, 5267–5277. 10.1128/JB.187.15.5267-5277.2005 16030221 PMC1196007

[B109] Parke J. L. Gurian-Sherman D. (2001). Diversity of the *Burkholderia cepacia* complex and implications for risk assessment of biological control strains. Annu. Rev. Phytopathol. 39, 225–258. 10.1146/annurev.phyto.39.1.225 11701865

[B110] Parrow N. L. Fleming R. E. Minnick M. F. (2013). Sequestration and scavenging of iron in infection. Infect. Immun. 81, 3503–3514. 10.1128/IAI.00602-13 23836822 PMC3811770

[B111] Peeters E. Sass A. Mahenthiralingam E. Nelis H. Coenye T. (2010). Transcriptional response of *Burkholderia cenocepacia* J2315 sessile cells to treatments with high doses of hydrogen peroxide and sodium hypochlorite. BMC Genomics 11:90. 10.1186/1471-2164-11-90 20137066 PMC2830190

[B112] Perez A. Issler A. C. Cotton C. U. Kelley T. J. Verkman A. S. Davis P. B. (2007). CFTR inhibition mimics the cystic fibrosis inflammatory profile. Am. J. Physiol. Lung Cell Mol. Physiol. 292, L383–L395. 10.1152/ajplung.00403.2005 16920886

[B113] Perumal Samy R. Stiles B. G. Sethi G. Lim L. H. K. (2017). Melioidosis: clinical impact and public health threat in the tropics. PLoS Negl. Trop. Dis. 11:e0004738. 10.1371/journal.pntd.0004738 28493905 PMC5426594

[B114] Pessi G. Braunwalder R. Grunau A. Omasits U. Ahrens C. H. Eberl L. (2013). Response of *Burkholderia cenocepacia* H111 to micro-oxia. PLoS ONE 8:e72939. 10.1371/journal.pone.0072939 24023794 PMC3759415

[B115] Ratledge C. Dover L. G. (2000). Iron metabolism in pathogenic bacteria. Annu. Rev. Microbiol. 54, 881–941. 10.1146/annurev.micro.54.1.881 11018148

[B116] Reid D. W. Anderson G. J. Lamont I. L. (2009). Role of lung iron in determining the bacterial and host struggle in cystic fibrosis. Am. J. Physiol. Lung Cell. Mol. Physiol. 297, L795–L802. 10.1152/ajplung.00132.2009 19700646

[B117] Reid D. W. Carroll V. O'may C. Champion A. Kirov S. M. (2007). Increased airway iron as a potential factor in the persistence of *Pseudomonas aeruginosa* infection in cystic fibrosis. Eur. Respir. J. 30, 286–292. 10.1183/09031936.00154006 17504792

[B118] Reid D. W. Lam Q. T. Schneider H. Walters E. H. (2004). Airway iron and iron-regulatory cytokines in cystic fibrosis. Eur. Respir. J. 24, 286–291. 10.1183/09031936.04.00104803 15332399

[B119] Reid D. W. Withers N. J. Francis L. Wilson J. W. Kotsimbos T. C. (2002). Iron deficiency in cystic fibrosis: relationship to lung disease severity and chronic *Pseudomonas aeruginosa* infection. Chest 121, 48–54. 10.1378/chest.121.1.48 11796431

[B120] Reik R. Spilker T. Lipuma J. J. (2005). Distribution of *Burkholderia cepacia complex* species among isolates recovered from persons with or without cystic fibrosis. J. Clin. Microbiol. 43, 2926–2928. 10.1128/JCM.43.6.2926-2928.2005 15956421 PMC1151955

[B121] Rhodes K. A. Schweizer H. P. (2016). Antibiotic resistance in *Burkholderia* species. Drug Resist. Update 28, 82–90. 10.1016/j.drup.2016.07.003 27620956 PMC5022785

[B122] Rose H. Baldwin A. Dowson C. G. Mahenthiralingam E. (2009). Biocide susceptibility of the *Burkholderia cepacia* complex. J. Antimicrob. Chemother. 63, 502–510. 10.1093/jac/dkn540 19153076 PMC2640157

[B123] Rottig M. Medema M. H. Blin K. Weber T. Rausch C. Kohlbacher O. (2011). NRPSpredictor2–a web server for predicting NRPS adenylation domain specificity. Nucleic Acids Res. 39, W362–W367. 10.1093/nar/gkr323 21558170 PMC3125756

[B124] Roy S. Douglas C. W. Stafford G. P. (2010). A novel sialic acid utilization and uptake system in the periodontal pathogen *Tannerella forsythia*. J. Bacteriol. 192, 2285–2293. 10.1128/JB.00079-10 20190043 PMC2863479

[B125] Runyen-Janecky L. J. (2013). Role and regulation of heme iron acquisition in gram-negative pathogens. Front. Cell. Infect. Microbiol. 3:55. 10.3389/fcimb.2013.00055 24116354 PMC3792355

[B126] Sanchez M. Sabio L. Galvez N. Capdevila M. Dominguez-Vera J. M. (2017). Iron chemistry at the service of life. IUBMB Life 69, 382–388. 10.1002/iub.1602 28150902

[B127] Sawana A. Adeolu M. Gupta R. S. (2014). Molecular signatures and phylogenomic analysis of the genus *Burkholderia*: proposal for division of this genus into the emended genus *Burkholderia* containing pathogenic organisms and a new genus *Paraburkholderia* gen. nov. harboring environmental species. Front. Genet. 5:429. 10.3389/fgene.2014.00429 25566316 PMC4271702

[B128] Schauer K. Rodionov D. A. De Reuse H. (2008). New substrates for TonB-dependent transport: do we only see the “tip of the iceberg”? Trends Biochem. Sci. 33, 330–338. 10.1016/j.tibs.2008.04.012 18539464

[B129] Schell M. A. Ulrich R. L. Ribot W. J. Brueggemann E. E. Hines H. B. Chen D.. (2007). Type VI secretion is a major virulence determinant in *Burkholderia mallei*. Mol. Microbiol. 64, 1466–1485. 10.1111/j.1365-2958.2007.05734.x 17555434

[B130] Shalom G. Shaw J. G. Thomas M. S. (2007). *In vivo* expression technology identifies a type VI secretion system locus in *Burkholderia pseudomallei* that is induced upon invasion of macrophages. Microbiology 153, 2689–2699. 10.1099/mic.0.2007/006585-0 17660433

[B131] Singh P. K. Parsek M. R. Greenberg E. P. Welsh M. J. (2002). A component of innate immunity prevents bacterial biofilm development. Nature 417, 552–555. 10.1038/417552a 12037568

[B132] Skaar E. P. (2010). The battle for iron between bacterial pathogens and their vertebrate hosts. PLoS Pathog. 6:e1000949. 10.1371/journal.ppat.1000949 20711357 PMC2920840

[B133] Smith E. E. Buckley D. G. Wu Z. Saenphimmachak C. Hoffman L. R. D'argenio D. A.. (2006). Genetic adaptation by *Pseudomonas aeruginosa* to the airways of cystic fibrosis patients. Proc. Natl. Acad. Sci. U.S.A. 103, 8487–8492. 10.1073/pnas.0602138103 16687478 PMC1482519

[B134] Smith M. D. Wuthiekanun V. Walsh A. L. White N. J. (1995). Quantitative recovery of *Burkholderia pseudomallei* from soil in Thailand. Trans. R. Soc. Trop. Med. Hyg. 89, 488–490. 10.1016/0035-9203(95)90078-0 8560518

[B135] Sokol P. A. (1986). Production and utilization of pyochelin by clinical isolates of *Pseudomonas cepacia*. J. Clin. Microbiol. 23, 560–562. 2937804 10.1128/jcm.23.3.560-562.1986 PMC268694

[B136] Sokol P. A. Woods D. E. (1988). Effect of pyochelin on *Pseudomonas cepacia* respiratory infections. Microb. Pathog. 5, 197–205. 10.1016/0882-4010(88)90022-8 3216778

[B137] Sokol P. A. Darling P. Woods D. E. Mahenthiralingam E. Kooi C. (1999). Role of ornibactin biosynthesis in the virulence of *Burkholderia cepacia*: characterization of pvdA, the gene encoding L-ornithine N(5)-oxygenase. Infect. Immun. 67, 4443–4455. 10456885 10.1128/iai.67.9.4443-4455.1999 PMC96763

[B138] Son M. S. Matthews W. J. Jr. Kang Y. Nguyen D. T. Hoang T. T. (2007). *In vivo* evidence of *Pseudomonas aeruginosa* nutrient acquisition and pathogenesis in the lungs of cystic fibrosis patients. Infect. Immun. 75, 5313–5324. 10.1128/IAI.01807-06 17724070 PMC2168270

[B139] Song E. Jaishankar G. B. Saleh H. Jithpratuck W. Sahni R. Krishnaswamy G. (2011). Chronic granulomatous disease: a review of the infectious and inflammatory complications. Clin. Mol. Allergy 9:10. 10.1186/1476-7961-9-10 21624140 PMC3128843

[B140] Song Y. Salinas D. Nielson D. W. Verkman A. S. (2006). Hyperacidity of secreted fluid from submucosal glands in early cystic fibrosis. Am. J. Physiol. Cell Physiol. 290, C741–C749. 10.1152/ajpcell.00379.2005 16207791

[B141] Sousa S. A. Ramos C. G. Leitao J. H. (2011). *Burkholderia cepacia* complex: emerging multihost pathogens equipped with a wide range of virulence factors and determinants. Int. J. Microbiol. 2011:607575. 10.1155/2011/607575 20811541 PMC2929507

[B142] Stephan H. Freund S. Beck W. Jung G. Meyer J. M. Winkelmann G. (1993). Ornibactins–a new family of siderophores from *Pseudomonas*. Biometals 6, 93–100. 10.1007/BF00140109 7689374

[B143] Stites S. W. Walters B. O'brien-Ladner A. R. Bailey K. Wesselius L. J. (1998). Increased iron and ferritin content of sputum from patients with cystic fibrosis or chronic bronchitis. Chest 114, 814–819. 10.1378/chest.114.3.814 9743172

[B144] Sun G. X. Zhou W. Q. Zhong J. J. (2006). Organotin decomposition by pyochelin, secreted by *Pseudomonas aeruginosa* even in an iron-sufficient environment. Appl. Environ. Microbiol. 72, 6411–6413. 10.1128/AEM.00957-06 16957273 PMC1563630

[B145] Tang A. C. Turvey S. E. Alves M. P. Regamey N. Tummler B. Hartl D. (2014). Current concepts: host-pathogen interactions in cystic fibrosis airways disease. Eur. Respir. Rev. 23, 320–332. 10.1183/09059180.00006113 25176968 PMC9487317

[B146] Thomas M. S. (2007). Iron acquisition mechanisms of the *Burkholderia cepacia* complex. Biometals 20, 431–452. 10.1007/s10534-006-9065-4 17295049

[B147] Thomas S. R. Ray A. Hodson M. E. Pitt T. L. (2000). Increased sputum amino acid concentrations and auxotrophy of *Pseudomonas aeruginosa* in severe cystic fibrosis lung disease. Thorax 55, 795–797. 10.1136/thorax.55.9.795 10950901 PMC1745865

[B148] Tuanyok A. Kim H. S. Nierman W. C. Yu Y. Dunbar J. Moore R. A.. (2005). Genome-wide expression analysis of iron regulation in *Burkholderia pseudomallei* and *Burkholderia mallei* using DNA microarrays. FEMS Microbiol. Lett. 252, 327–335. 10.1016/j.femsle.2005.09.043 16242861

[B149] Tunney M. M. Field T. R. Moriarty T. F. Patrick S. Doering G. Muhlebach M. S.. (2008). Detection of anaerobic bacteria in high numbers in sputum from patients with cystic fibrosis. Am. J. Respir. Crit. Care Med. 177, 995–1001. 10.1164/rccm.200708-1151OC 18263800

[B150] Tyrrell J. Whelan N. Wright C. Sa-Correia I. McClean S. Thomas M.. (2015). Investigation of the multifaceted iron acquisition strategies of *Burkholderia cenocepacia*. Biometals 28, 367–380. 10.1007/s10534-015-9840-1 25725797

[B151] Uehlinger S. Schwager S. Bernier S. P. Riedel K. Nguyen D. T. Sokol P. A.. (2009). Identification of specific and universal virulence factors in *Burkholderia cenocepacia* strains by using multiple infection hosts. Infect. Immun. 77, 4102–4110. 10.1128/IAI.00398-09 19528212 PMC2738042

[B152] Valvano M. A. (2015). Intracellular survival of *Burkholderia cepacia* complex in phagocytic cells. Can. J. Microbiol. 61, 607–615. 10.1139/cjm-2015-0316 26220706

[B153] Vargas-Straube M. J. Camara B. Tello M. Montero-Silva F. Cardenas F. Seeger M. (2016). Genetic and functional analysis of the biosynthesis of a non-ribosomal peptide siderophore in *Burkholderia xenovorans* LB400. PLoS ONE 11:e0151273. 10.1371/journal.pone.0151273 26963250 PMC4786211

[B154] Vergunst A. C. Meijer A. H. Renshaw S. A. O'callaghan D. (2010). *Burkholderia cenocepacia* creates an intramacrophage replication niche in zebrafish embryos, followed by bacterial dissemination and establishment of systemic infection. Infect. Immun. 78, 1495–1508. 10.1128/IAI.00743-09 20086083 PMC2849400

[B155] Verma A. K. Saminathan M. Neha Tiwari R. Dhama K. Vir Singh S. (2014). Glanders-A re-emerging zoonotic disease: a review. J. Biol. Sci. 14, 38–51. 10.3923/jbs.2014.38.51

[B156] Visca P. Ciervo A. Sanfilippo V. Orsi N. (1993). Iron-regulated salicylate synthesis by *Pseudomonas* spp. J. Gen. Microbiol. 139, 1995–2001. 10.1099/00221287-139-9-1995 7504066

[B157] Visca P. Colotti G. Serino L. Verzili D. Orsi N. Chiancone E. (1992). Metal regulation of siderophore synthesis in *Pseudomonas aeruginosa* and functional effects of siderophore-metal complexes. Appl. Environ. Microbiol. 58, 2886–2893. 1444402 10.1128/aem.58.9.2886-2893.1992 PMC183023

[B158] Visser M. B. Majumdar S. Hani E. Sokol P. A. (2004). Importance of the ornibactin and pyochelin siderophore transport systems in *Burkholderia cenocepacia* lung infections. Infect. Immun. 72, 2850–2857. 10.1128/IAI.72.5.2850-2857.2004 15102796 PMC387874

[B159] Wand M. E. Muller C. M. Titball R. W. Michell S. L. (2011). Macrophage and *Galleria mellonella* infection models reflect the virulence of naturally occurring isolates of *B. pseudomallei, B. thailandensis* and *B. oklahomensis*. BMC Microbiol. 11:11. 10.1186/1471-2180-11-11 21241461 PMC3025829

[B160] Wang J. Lory S. Ramphal R. Jin S. (1996). Isolation and characterization of *Pseudomonas aeruginosa* genes inducible by respiratory mucus derived from cystic fibrosis patients. Mol. Microbiol. 22, 1005–1012. 10.1046/j.1365-2958.1996.01533.x 8971720

[B161] Whitby P. W. Vanwagoner T. M. Springer J. M. Morton D. J. Seale T. W. Stull T. L. (2006). *Burkholderia cenocepacia* utilizes ferritin as an iron source. J. Med. Microbiol. 55, 661–668. 10.1099/jmm.0.46199-0 16687582

[B162] White N. J. (2003). Melioidosis. Lancet 361, 1715–1722. 10.1016/S0140-6736(03)13374-0 12767750

[B163] Whitlock G. C. Estes D. M. Torres A. G. (2007). Glanders: off to the races with *Burkholderia mallei*. FEMS Microbiol. Lett. 277, 115–122. 10.1111/j.1574-6968.2007.00949.x 18031330

[B164] Wiersinga W. J. Currie B. J. Peacock S. J. (2012). Melioidosis. N. Engl. J. Med. 367, 1035–1044. 10.1056/NEJMra1204699 22970946

[B165] Wiersinga W. J. Van Der Poll T. White N. J. Day N. P. Peacock S. J. (2006). Melioidosis: insights into the pathogenicity of *Burkholderia pseudomallei*. Nat. Rev. Microbiol. 4, 272–282. 10.1038/nrmicro1385 16541135

[B166] Wolpert M. Gust B. Kammerer B. Heide L. (2007). Effects of deletions of mbtH-like genes on clorobiocin biosynthesis in *Streptomyces coelicolor*. Microbiology 153, 1413–1423. 10.1099/mic.0.2006/002998-0 17464055

[B167] Worlitzsch D. Tarran R. Ulrich M. Schwab U. Cekici A. Meyer K. C.. (2002). Effects of reduced mucus oxygen concentration in airway *Pseudomonas* infections of cystic fibrosis patients. J. Clin. Invest. 109, 317–325. 10.1172/JCI0213870 11827991 PMC150856

[B168] Yabuuchi E. Kosako Y. Oyaizu H. Yano I. Hotta H. Hashimoto Y.. (1992). Proposal of *Burkholderia* gen. nov. and transfer of seven species of the genus *Pseudomonas* homology group II to the new genus, with the type species *Burkholderia cepacia* (Palleroni and Holmes 1981) comb. nov. Microbiol. Immunol. 36, 1251–1275. 10.1111/j.1348-0421.1992.tb02129.x 1283774

[B169] Yang H. M. Chaowagul W. Sokol P. A. (1991). Siderophore production by *Pseudomonas pseudomallei*. Infect. Immun. 59, 776–780. 1825486 10.1128/iai.59.3.776-780.1991 PMC258326

[B170] Yang H. Kooi C. D. Sokol P. A. (1993). Ability of *Pseudomonas pseudomallei* malleobactin to acquire transferrin-bound, lactoferrin-bound, and cell-derived iron. Infect. Immun. 61, 656–662. 7678587 10.1128/iai.61.2.656-662.1993 PMC302777

[B171] Yoder-Himes D. R. Konstantinidis K. T. Tiedje J. M. (2010). Identification of potential therapeutic targets for *Burkholderia cenocepacia* by comparative transcriptomics. PLoS ONE 5:e8724. 10.1371/journal.pone.0008724 20090946 PMC2806911

[B172] Yoon S. S. Coakley R. Lau G. W. Lymar S. V. Gaston B. Karabulut A. C.. (2006). Anaerobic killing of mucoid *Pseudomonas aeruginosa* by acidified nitrite derivatives under cystic fibrosis airway conditions. J. Clin. Invest. 116, 436–446. 10.1172/JCI24684 16440061 PMC1350997

[B173] Youard Z. A. Wenner N. Reimmann C. (2011). Iron acquisition with the natural siderophore enantiomers pyochelin and enantio-pyochelin in *Pseudomonas* species. Biometals 24, 513–522. 10.1007/s10534-010-9399-9 21188474

[B174] Yuhara S. Komatsu H. Goto H. Ohtsubo Y. Nagata Y. Tsuda M. (2008). Pleiotropic roles of iron-responsive transcriptional regulator Fur in *Burkholderia multivorans*. Microbiology 154, 1763–1774. 10.1099/mic.0.2007/015537-0 18524931

[B175] Zeng X. Mo Y. Xu F. Lin J. (2013). Identification and characterization of a periplasmic trilactone esterase, Cee, revealed unique features of ferric enterobactin acquisition in *Campylobacter*. Mol. Microbiol. 87, 594–608. 10.1111/mmi.12118 23278903 PMC3556234

[B176] Zlosnik J. E. Zhou G. Brant R. Henry D. A. Hird T. J. Mahenthiralingam E.. (2015). *Burkholderia* species infections in patients with cystic fibrosis in British Columbia, Canada. 30 years' experience. Ann. Am. Thorac. Soc. 12, 70–78. 10.1513/AnnalsATS.201408-395OC 25474359

