# Corrigendum: Iron Acquisition Mechanisms and Their Role in the Virulence of *Burkholderia* Species

**DOI:** 10.3389/fcimb.2018.00305

**Published:** 2018-08-24

**Authors:** Aaron T. Butt, Mark S. Thomas

**Affiliations:** Department of Infection, Immunity and Cardiovascular Disease, Faculty of Medicine, Dentistry and Health University of Sheffield, Sheffield, United Kingdom

**Keywords:** *Burkholderia*, iron, siderophores, haem uptake, cystic fibrosis, melioidosis

In the original article, there was a mistake in the legend for **Figure 2** as published. Ornibactins and malleobactins contain two hydroxamates groups, not one, and a single α-hydroxycarboxylate group, not two β-hydroxycarboxylate groups. The correct legend appears below. The authors apologize for this error and state that this does not change the scientific conclusions of the article in any way.

**Figure 2**: Structure of siderophores produced by *Burkholderia* species. **(A)** Ornibactins contain an N-terminal ornithine that is acylated with a C4, C6, or C8 β-hydroxycarboxylic acid on the δ-amino nitrogen atom, giving rise to ornibactin-C4, -C6, or -C8. The δ-amino nitrogen atom is also hydroxylated. The other three amino acids in the tetrapeptide are D-hydroxyaspartate, L-serine, and the C-terminal ornithine that is formylated and hydroxylated on the δ-amino nitrogen atom and the carboxyl group is conjugated to putrescine. As with the malleobactins, they contain two bidentate hydroxamate ligands and a single bidentate α-hydroxycarboxylate ligand. **(B)** Malleobactin E, the siderophore-active malleobactin congener of *B. thailandensis*. **(C)** The siderophore-active malleobactin congener of *B. xenovorans*, tentatively referred to here as “malleobactin X.” **(D)** Cepaciachelin contains two 2,3-DHBA groups that form amide linkages with the two amino groups of lysine, which in turn is conjugated to a molecule of putrescine (1,4-diaminobutane) on its α-carboxyl group. **(E)** Pyochelin contains two less commonly occurring bidentate iron-chelating groups (2-hydroxyphenyl thiazoline and N-methylthiazolidine-4-carboxylate). **(F)** Cepabactin, a cyclic hydroxamate bidentate siderophore. Chemical groups that distinguish the ornibactins and malleobactins are indicated in red circles or ellipses.

The original article has been updated.

## Conflict of interest statement

The authors declare that the research was conducted in the absence of any commercial or financial relationships that could be construed as a potential conflict of interest.

