# Supplementary material for: Iron Acquisition Mechanisms and Their Role in the Virulence of Burkholderia Species
Source: Front Cell Infect Microbiol. 2017 Nov 6;7:460. doi: 10.3389/fcimb.2017.00460 (PMC5681537; doi:10.3389/fcimb.2017.00460)
Supplement: Supplementary file 1 [file Table1.DOCX]

**Supplementary Table 1. Ornibactin, malleobactin and phymabactin gene loci**

**Siderophore Species Strain Locus^a^ Old locus^a^**

Ornibactin

*B. ambifaria* AMMD BAMB_RS07735-BAMB_RS07805 Bamb_1529-Bamb_1543

*B. anthina* AZ-4-2-10-S1-D7 WS64_RS06915-WS64_RS06985 WS64_06915-WS64_06985

*B. cenocepacia* J2315 QU43_RS44960-QU43_RS45030 BCAL1688-BCAL1702

*B. cepacia* GG4 GEM_RS08865-GEM_RS08795 GEM_1784-GEM_1770

*B. contaminans* MS14 NL30_RS14930-NL30_RS14860 NL30_14935-NL30_14865

*B. diffusa* RF2-non-BP9 WI26_RS07780-WI26_RS07850 WI26_RS07780-WI26_RS07850

*B. dolosa* AU0158 AK34_RS20320-AK34_RS20250 AK34_1468-AK34_1454

*B. lata* 383 BCEP18194_RS14010-BCEP18194_RS14080 Bcep18194_A4778-Bcep18194_A4792

*B. latens* AU17928 WK25_RS08035-WK25_RS08105 WK25_08030-WK25_08100

*B. metallica* FL-6-5-30-S1-D7 WJ16_RS08190-WJ16_RS08260 WJ16_08180-WJ16_08260

*B. multivorans*^b^ ATCC 17616 BMULJ_RS08125-BMULJ_RS08195 BMULJ_01637-BMULJ_01651

BMUL_RS08205-BMUL_RS08135 Bmul_1606-Bmul_1592

*B. paludis*^c^ MSh1 GQ56_0110815-GQ56_0110850^c^ n/a

*B. pseudomultivorans* SUB-INT23-BP2 WS57_RS26685-WS57_RS26770 WS57_26630-WS57_26715

*B. pyrrocinia* 2327 ABD05_RS13980-ABD05_RS14050 ABD05_13995-ABD05_14065

*B. seminalis* FL-5-4-10-S1-D7 WJ12_RS08100-WJ12_RS08170 WJ12_08090-WJ12_08160

*B. stabilis* ATCC BAA-67 BBJ41_RS03900-BBJ41_RS03830 BBJ41_03900-BBJ41_03830

*B. stagnalis* MSMB735 WT74_RS08435-WT74_RS08510 WT74_08420-WT74_08495

*B. territorii* RF8-non-BP5 WS51_RS18700-WS51_RS18770 WS51_18690-WS51_18760

*B. ubonensis* MSMB22 BW23_RS15460-BW23_RS15390 BW23_96-BW23_82

*B. ubonensis* RF23-BP41 WI31_RS12785-WI31_RS12865 WI31_12775-WI31_12855

*B. vietnamiensis* G4 Bcep1808_1577 -Bcep1808_1590 n/a

‘Bcc’ ATCC 31433 B7P44_RS08445-B7P44_RS08520 n/a

Malleobactin

*B. mallei* ATCC 23344 BMA1191-BMA1177 n/a

*B. phytofirmans* PsJN BPHYT_RS20215-BPHYT_RS20280 Bphyt_4063-Bphyt_4076

*B. pseudomallei* K96243 BPSL1787-BPSL1774 n/a

*B. thailandensis* E264 BTH_RS24590-BTH_RS24525 BTH_I2427-BTH_I2414

*B. xenovorans* LB400 BXE_RS34310-BXE_RS34255 Bxe_B0517-Bxe_B0528

*C. fungivorans* Ter331 CFU_RS11880-CFU_RS11820 CFU_2304-CFU_2292

Phymabactin

*B. caribensis* MWAP64 AN416_RS21305-AN416_RS21240 AN416_21295-AN416_21230

*B. phymatum* STM815 BPHY_RS20390-BPHY_RS20330 Bphy_4047-Bphy_4035

*B. terrae*^d^ BS001 WQE_04287-WQE_04317/ n/a

WQE_26310-WQE_26340 n/a

^a^Gene loci refer to the first and last genes in the corresponding gene cluster as shown in Figure 3.

^b^Two alternative annotations are shown for *B. multivorans* ATCC 17616.

^c^Genes shown encode the ornibactin transport system and the sigma factor regulator of ornibactin gene transcription but not the ornibactin biosynthesis enzymes.

^d^Gene loci are present on two separate sequence contigs (contig00031 and contig00149) at the time of constructing this table.
