# Supplementary material for: Iron Acquisition Mechanisms and Their Role in the Virulence of Burkholderia Species
Source: Front Cell Infect Microbiol. 2017 Nov 6;7:460. doi: 10.3389/fcimb.2017.00460 (PMC5681537; doi:10.3389/fcimb.2017.00460)
Supplement: Supplementary file 2 [file Table2.DOCX]

**Supplementary Table 2. Cepaciachelin gene loci**

**Species Strain Locus^a^ Old locus^a^**

*B. ambifaria* AMMD BAMB_RS08485-BAMB_RS08545 Bamb_1684-Bamb_1696

*B. metallica* FL-6-5-30-S1-D7 WJ16_RS08990-WJ16_RS09055 WJ16_08975-WJ16_09040

*B. multivorans*^b^ CGD1 BURMUCGD1_1525-BURMUCGD1_1512 n/a

*B. pseudomultivorans*^c^ MSMB368 WT56_00605-WT56_00670 n/a

*B. pyrrocinia* 2327 ABD05_RS14905-ABD05_RS14970 ABD05_14915-ABD05_14980

*B. stagnalis* MSMB735 WT74_RS07910-WT74_RS07845 WT74_07895-WT74_07830

*B. ubonensis* RF23-BP41 WI31_RS12350-WI31_RS12285 WI31_12340-WI31_12275

‘Bcc’ ATCC 31433 B7P44_RS07860-B7P44_RS07795 n/a

^a^Gene loci refer to the first and last genes (*cpcH* and *cpcJ*, respectively) in the cepaciachelin gene cluster shown in Figure 4.

^b^The *B. multivorans* type strain ATCC 17616 does not contain the cepaciachelin gene cluster.

^c^The cepaciachelin gene cluster is present in *B. pseudomultivorans* strain MSMB368 but not in other strains currently in the database.
