# Supplementary material for: Iron Acquisition Mechanisms and Their Role in the Virulence of Burkholderia Species
Source: Front Cell Infect Microbiol. 2017 Nov 6;7:460. doi: 10.3389/fcimb.2017.00460 (PMC5681537; doi:10.3389/fcimb.2017.00460)
Supplement: Supplementary file 3 [file Table3.DOCX]

**Supplementary Table 3. Pyochelin gene loci**

**Species Strain Locus^a^ Old locus^a^**

*B. anthina* MSMB1496 WT13_02015-WT13_02080 n/a

*B. cenocepacia* J2315 QU43_RS65475-QU43_RS65540 BCAM2221-BCAM2235

*B. cepacia*^b^ ATCC 25416 APZ15_RS27950-APZ15_RS27885 APZ15_27950-APZ15_27885

*B. lata* 383 BCEP18194_RS26180-BCEP18194_RS26115 Bcep18194_B0680-Bcep18194_B0667

*B. paludis* MSh1 GQ56_0111185-GQ56_0111120 n/a

*B. seminalis* FL-5-4-10-S1-D7 WJ12_RS31035-WJ12_RS31100 WJ12_31015-WJ12_31080

*B. stabilis* ATCC BAA-67 BBJ41_RS24835-BBJ41_RS24900 BBJ41_24835-BBJ41_24900

*B. pseudomallei* K96243 BPSS0594-BPSS0581 n/a

^a^Gene loci refer to the first and last genes (*fptX* and *pchA*, respectively) in the pyochelin gene cluster shown in Figure 4.

^b^*B. cepacia* ATCC 25416 (the type strain) produces pyochelin whereas strain GG4 does not encode the capacity to produce this siderophore.
