# Supplementary material for: Iron Acquisition Mechanisms and Their Role in the Virulence of Burkholderia Species
Source: Front Cell Infect Microbiol. 2017 Nov 6;7:460. doi: 10.3389/fcimb.2017.00460 (PMC5681537; doi:10.3389/fcimb.2017.00460)
Supplement: Supplementary file 4 [file Table4.DOCX]

**Supplementary Table 4. Haem uptake genetic loci of pathogenic *Burkholderia* species**

**Species Strain Locus^a^ Old locus^a^**

*B. arboris^b^* ? ?

*B. ambifaria* AMMD BAMB_RS23890-BAMB_RS23910 Bamb_4772-Bamb_4776

*B. anthina* AZ-4-2-10-S1-D7 WS64_27095-WS64_27075 n/a

*B. cenocepacia* J2315 QU43_RS67585-QU43_RS67605 BCAM2626-BCAM2630

*B. cepacia* ATCC 25416 APZ15_RS23630-APZ15_RS23650 APZ15_23630-APZ15_23650

*B. contaminans* MS14 NL30_RS16925-NL30_RS16945 NL30_16920-NL30_16940

*B. diffusa*^c^ MSMB866 WI71_09210-WI71_09190 n/a

*B. dolosa* AU0158 AK34_RS00410-AK34_RS00390 AK34_3247-AK34_3243

*B. lata* 383 BCEP18194_RS23870-BCEP18194_RS23850 Bcep18194_B0221-Bcep18194_B0217

*B. latens*^d^ AU17928 WK25_28740-WK25_28760 n/a

*B. metallica* FL-6-5-30-S1-D7 WJ16_RS31540-WJ16_RS31560 WJ16_31510-WJ16_31530

Bcc

*B. multivorans*^e^ ATCC 17616 BMUL_RS16900-BMUL_RS16880 Bmul_3338-Bmul_3334

BMULJ_RS25905-BMULJ_RS25925 BMULJ_05192-BMULJ_05188

*B. paludis* MSh1 GQ56_0128135-GQ56_0128155 n/a

*B. pseudomultivorans* SUB-INT23-BP2 WS57_RS01155-WS57_RS01135 WS57_01155-WS57_01135

*B. pyrrocinia* 2327 (DSM10685) ABD05_RS22580-ABD05_RS22560 ABD05_22580-ABD05_22560

*B. seminalis* FL-5-4-10-S1-D7 WJ12_33030-WJ12_33050 n/a

*B. stabilis* ATCC BAA-67 BBJ41_RS27015-BBJ41_RS27035 BBJ41_27015-BBJ41_27035

*B. stagnalis*^f^ MSMB735 WT74_RS31830-WT74_RS31855 WT74_31785-WT74_31810

*B. territorii* RF8-non-BP5 WS51_RS09890-WS51_RS09910 WS51_09890-WS51_09910

*B. ubonensis* MSMB22 BW23_RS02620-BW23_RS02640 BW23_3819-BW23_3823

*B. vietnamiensis*^g^ G4 n/a n/a

*B. mallei* ATCC 23344 BMAA1826-BMAA1830 n/a

*B. pseudomallei* K96243 BPSS0244-BPSS0240 n/a

*B. gladioli*^h^ BSR3 BGLA_RS01200- BGLA_RS01210 bgla_1g02490-bgla_1g02510

BGLA_RS33815-BGLA_RS33820 bgla_2g28390-bgla_2g28400

^a^Gene loci refer to the first and last genes (*bhuR* and *bhuV*, respectively) in the *bhuRSTUV* haem uptake gene cluster of representative

pathogenic *Burkholderia* species as shown in Figure 4.

^b^A *B. arboris* genome sequence is not currently available to interrogate.

^c^The *bhu* gene cluster is inactivated in *B. diffusa* strain RF2-non-BP9.

^d^The *bhuU* gene in *B. latens* contains multiple two codon repeats at the beginning of the ORF. It is annotated as a pseudogene but may be functional.

^e^Two alternative annotations are shown for *B. multivorans* ATCC 17616.

^f^The *B. stagnalis* haem uptake operon contains an additional gene encoding a VOC family metalloenzyme located between *bhuU* and *bhuV*.

^g^*B. vietnamiensis* does not contain the *bhu* gene cluster.

^h^The *B. gladioli bhuRSTUV* genes are organised into two operons located on separate chromosomes (see text for details).

n/a, not applicable
